# Adaptive differential current relay based on form/ripple factors for busbar current signals

**DOI:** 10.1038/s41598-025-12832-8

**Published:** 2025-08-12

**Authors:** R. A. Mahmoud

**Affiliations:** Department of Electrical Power and Machines Engineering (PME), College of Engineering Science & Technology, Misr University for Science and Technology (MUST), 6Th of October City, Giza Egypt

**Keywords:** Fault currents, CT saturation, DC component, Differential current relays, Adaptive relays, Form factor, Ripple factor, Energy science and technology, Engineering

## Abstract

This paper presents an adaptive protection algorithm that adjusts the tripping characteristics of the differential relaying schemes in response to changes in CT saturation levels and DC component content of fault currents. Moreover, the main protection function differentiates between internal and external faults with or without CT saturation. This is accomplished by estimating the appropriate tripping characteristic slope using form and ripple factors calculated for the current signals measured at the entering and exiting terminals of the protected equipment. The modified approach aims to achieve automatic resetting to inhibit the relay operation during external faults and to avoid any delay or restriction of the relay operation during severe internal faults due to the presence of harmonics. Numerous cases, including various types of internal and external faults with or without CTs saturation and DC component, are carried out on a typical power system simulated using the ATP platform. The proposed algorithm can be performed utilizing the MATLAB software, which can receive current measurements from the ATP simulator. The simulation results manifest the functional efficiency of the suggested technique under diverse operating and fault conditions and its ability to discriminate fault location. Additionally, the response time of the suggested technique is roughly 10.0 ms in the event of internal faults, which is also appropriate for preventing the technique from functioning in the case of non-fault or external fault disturbances. Furthermore, it is able to recognize CT saturation conditions, assess the degree of current distortion, and select which feeder CT is saturated. Besides, the outcomes demonstrate the extreme simplicity, effectiveness, stability, accuracy, and reliability of the proposed algorithm. Quantitative findings from the extensive case studies indicate that the estimated ratios of the protection’s accuracy, dependability, security, and reliability are greater than 99.40%. It is applicable to Smart Grids (SGs) and Substation Automation Systems (SAS), as the algorithm is one of the applications in digital protection relays/systems.

**Supplementary Information:**

The online version contains supplementary material available at 10.1038/s41598-025-12832-8.

## Introduction

Power system components require continuous monitoring and a fast protection response. The major components of the power system include synchronous generators, power transformers, large motors, feeders, and busbars. The objective of the protection is to isolate the component that is susceptible to the fault event in order to prevent any damage to the power system^1^. A differential current relay is essential for fast tripping the protected component, which can sense the disparity between phase currents entering and leaving the component^1^. The biased or percentage differential current relay is the most prevalent type of differential relays that distinguishes between differential and restraining currents^2,3^. If an imbalance is found between the two quantities with a presetting value, the relay will activate and trip all circuit breakers of the protected equipment^4^. It is the main protection for power systems because it operates instantaneously, protects the equipment against both earth and phase faults, and has a limited protection zone^5^. Even though the differential relay is considered to be reliable and robust, it faces many drawbacks due to nonlinear operation resulting from variations in CT characteristics, CT ratios, and fault currents accompanied by DC components^6^. When there is variation in the through fault current due to system configuration changes, the relay may give reduced accuracy. Hence, if the maximum through fault current is accurately determined, a proper characteristic slope can be selected, and then the relay malfunction will be avoided^7^. Conventional percentage differential relays may lose their selectivity property in the instances of external fault with a significant amount of DC component due to the high value of the decaying time constant, which may result in over-tripping if any CT is saturated^8^. Moreover, they do not operate under the low internal fault current as expected. The large quantity of fault currents may cause CT saturation within the first half-cycle or the full-cycle following fault time inception ^9^.

### Motivation and incitement

Most traditional differential relays typically possess fixed-bias tripping characteristics. In some situations of severe CT saturation, the selection of an unsuitable restraint factor may cause the relay to malfunction^10^. Adaptive protection relays/systems can change relay settings online using input signals measured from local transducers or a central control system. Therefore, certain protection shortcomings can be addressed by the adaptive protections, such as (1) the relay stability during large external faults with CT saturation conditions, (2) the relay sensitivity during low internal faults, and (3) the presence of harmonics may delay or prevent relay operation in the event of severe internal faults^11^.

### Literature review

Numerous numerical techniques were presented in the field of adaptive differential protection systems, which were used to protect various electrical elements in conventional and smart grids. Several methods based on statistical/mathematical models were employed, encompassing but not limited to alienation coefficients^12,13^, correlation coefficients^14^ transient signal analysis^15^, wireless communication and computer technologies^16^, alpha plane^17^, machine learning^18^, superimposed sequence current^19^, superimposed reactive energy^20^, limited wide area^21^, Artificial Neural Networks (ANNs)^22^, Fuzzy decision and graph^23^, Wavelet Transform (WT) integrated with correlation analysis^24^, Continuous Wavelet Transform (CWT)^25^, and Phasor Measurement Unit (PMU)^26^. In^27^, a protection scheme based on analytically derived pick-up and slope of the differential relay characteristic was presented, where its sensitivity was enhanced during internal faults using input information like rating and connection of CTs. These methods aim to ensure a proper operation and improve the protection performance, stability, sensitivity, security, accuracy and efficiency. Although some of these methods are complicated, their use is beneficial^23–25^. Three critical issues for a conventional differential overcurrent relay in^28^ are addressed in this study, which can be listed as follows:

1. the sensitivity and security of the relay cannot be automatically modified utilizing the prescribed low pickup differential current and the selected single restraining factor that identify the tripping and blocking zones of the operating characteristic,
2. The presence of harmonics in the event of severe internal faults will accelerate the relay operation, which originates from the DC components and CT saturation, and.
3. The relay needs a supplementary algorithm or device to define the event of external faults with CT saturation extent.

As a consequence, the implementation of an adaptation strategy for tripping characteristics is indispensable to enhance the relay stability in the instance of external faults with CT saturation.

In^29^, the adaptive overcurrent relay (AOCR) used the three Z-scores of the three single phase current signals (measured in the distribution system with/without Fault Current Limiters (FCLs) or with/without Distributed Generators (DGs)) to select the faulty phase and define the fault type. Also, the FCLs limit the fault current magnitude and prevent the presence of CT saturation; nevertheless, the relay couldn’t identify the fault location, whether it is internal or external^29^. In^30^, the adaptive overcurrent relay (AOCR) was contingent on Fano Factors (FFs) of current signals. It was also unable to ascertain the fault protection zone^30^. In^31^, an adaptive protection strategy for overcurrent relays, meticulously tailored to accommodate the fluctuations in electrical load, was presented. This strategy used a hybrid algorithm, which amalgamated the strengths of three preeminent metaheuristic models: Improved Harmony Search, Particle Swarm Optimization, and Differential Evolution^31^. The results and analyses substantiated the efficacy of the algorithm in optimizing the coordination among overcurrent relays aiming to uphold the overarching protective imperatives of the grid; moreover, the operation speed of the relay was reasonably high^31^.

In^32^, a virtual consensus-based wide area differential protection (WADP) method through cooperative control concepts and graph theory was presented. It was designed to adapt to network topology changes using the existing telecommunication connections. By implementing dynamic decision-making within protection areas, the method^32^ offered an efficient and high-speed solution to power grid protection. While, the paper^32^ primarily focused on applying the proposed virtual differential protection to support other distance protections. In^33^, a dynamic wide-area cooperative protection based on cooperative control of distributed multi-agent systems and graph theoretical methods was introduced. The protection decisions were collective, while a centralized master did not exist, and local information was applied. A fault detection code and a wide-area fault detection code were used for fault detection^33^. The suggested protection performance was studied as a backup protection for distance relays during normal, stable power swings, unstable power swings, and load encroachment conditions^33^. In^34^, the study used the slope degree of grey incidence analysis model to design a methodology for busbar protection. A grey-based busbar protection criterion was established to inspect the similarities of the superimposed currents measured by nearby CTs inside the busbar protection zone. It was seen that the methodology^34^ could reliably discriminate between internal and external faults. Moreover, the results demonstrated that the protection indexes of the scheme^34^ are satisfactory for different conditions, including fault type, high fault resistance, fault, simultaneous faults, and faults with CT saturation. In^35^, an integrated busbar protection scheme based on a coherence approach was applied. The approach^35^ had the ability to discern the faulty phase and determine the fault type and fault protection zone; besides, it could detect the case of external faults with CT saturation extent.

### Contribution and paper organization

This work develops an adaptive protection mechanism that manages the tripping features of differential relay responses to change in the CT saturation levels and the content of the DC components during the current faults. The approach depends on evaluating the most convenient slope for the relay tripping characteristics by calculating the form and ripple factors of the current signals in order to finally formulate a restraint signal making the relay operation more appropriate when facing the external faults. Moreover, the estimated form and ripple factors are useful for accelerating the relay operation in the incidence of large internal fault currents with high DC components. These features enable the technique to reinforce the protection stability and security during periods of CT saturation and DC components of fault currents. Besides, its main function is to select the faulty phase(s), classify the faults, and discriminate the fault location. A typical three-phase power network is subject to a diverse range of internal and external faults, including but not limited to CT saturation and varying operating conditions. This proposal possesses the following characteristics:

1. The algorithm is able to identify steady-state conditions, and faults located on the protected component.
2. The instances of internal and external shunt faults can be swiftly differentiated,
3. The approach can be used to discriminate between external shunt faults with or without CT saturation occurrence,
4. The technique can be used to categorize fault types, select faulty phases, and discriminate fault directions,
5. The approach is able to detect CT saturation conditions and assess the extent of DC components,
6. The convenient tripping zone of the differential current protection can be neatly determined using novel mathematical models based on form and ripple factors computed for current signals of the protected component,
7. The restraining slope of the differential current relay can be automatically adjusted when the level of CT saturation or the amount of DC components changes,
8. The algorithm can be employed online and is compatible with other digital differential current relays made by different companies,
9. Local current measurements can only be used, reducing the time spent to transmit and process the local data,
10. This approach is suitable for both conventional and smart grids with a variety of voltage and power ratings,
11. The methodology can be harnessed in practice,
12. The method maintains protection stability despite the specifications of power element parameters are changed,
13. The algorithm can be applied to protect a single-phase or three-phase component or equipment,
14. The approach responds when internal faults occur, while its operation is blocked when the protected element is healthy or when external faults is existent,
15. The data window and the specified settings of the differential current algorithm can be used to alter the sensitivity and speed of fault detection, and
16. The proposed algorithm can be characterized as being smart, reliable, swift, stable, and precise,

This paper is arranged as follows: Section “Traditional biased differential relay” describes the applications, the principle of operation, and the tripping characteristics of the conventional biased differential relay made by the Siemens Company. Mathematical models and algorithm procedure for the adaptive differential current relay are elaborately explained in Section “Proposed method”. A simulation power system under study and the specifications of its components are exhibited in Section “Power system description”, which is used to validate the performance of the advanced algorithm. The simulation outcomes are described and analyzed in Section “Simulations and results analysis”. In Section “Protection scheme assessment”, the benefits of the adaptive differential current protection are enumerated, as well as a thorough comparison between the current proposed and recent published methods. Eventually, the results are concluded in Section “Conclusions”.

## Traditional biased differential relay

In order to present a proposal to improve the stability of differential relay characteristics during periods of CTs saturation and DC component extent, one model of biased differential current relays (made by Siemens Company) should be described^28^.

### Description and application

The SIPROTEC 7SS60 system is one sample of numerical differential current relays used to protect busbars. It is compatible with a wide variety of busbar configurations, and is suitable for all voltage levels. This relay is designed for diverse busbars, such as single, one and half breaker and double busbars with or without bus couplers^28^.

### Function principle

The 7SS60 protection system operates with the differential current measuring principle to protect busbars. Its algorithm relies on Kirchhoff’s current law, which states that in a fault-free condition, the vector sum (*I*_*d*_) of all currents flowing into an independent busbar section must be zero. Some minor deviations from this law may be attributed to errors in CTs, inaccuracies in the matching of the CTs ratios and measurement inaccuracies. Further errors may be due to CTs saturation in the presence of large external short-circuit currents, which are counteracted by a load-dependent supplementary restraint^28^. The restraint current (*I*_*r*_) is derived from the load condition, which arises from the summation of all the busbar current magnitudes^28^.

### Differential relay tripping characteristics

**Fig. 1:**
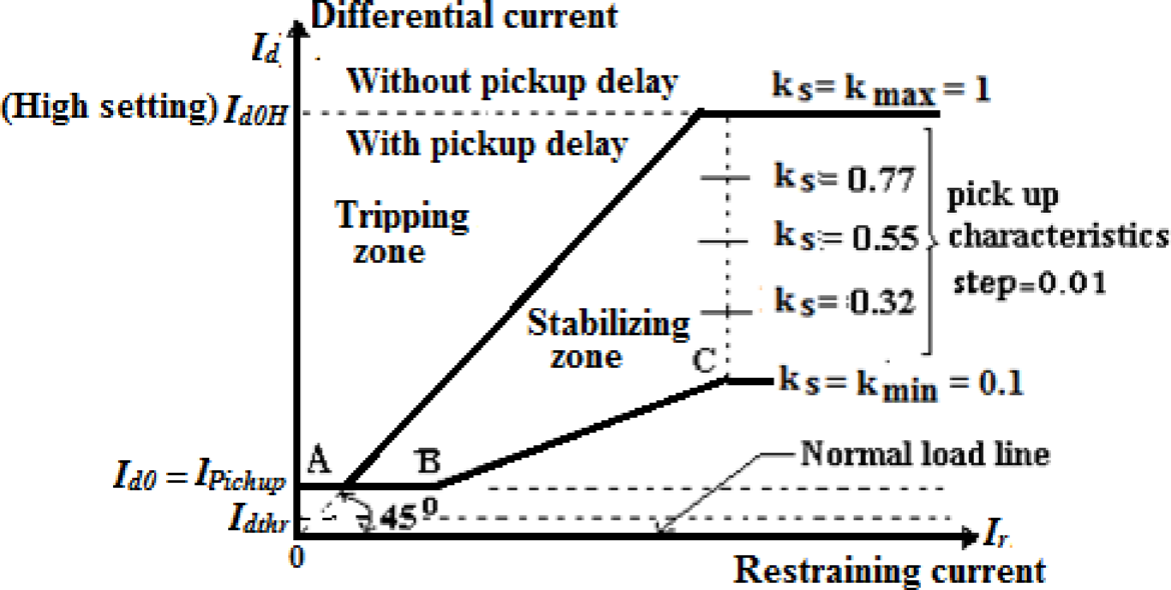
Tripping characteristics of the 7SS60 differential protection relay.

The tripping characteristic settings of the differential relay encompass a pickup value (*I*_*d0*_) and a restraint factor (*K*_*s*_) that considers both linear and non-linear CT errors. Tripping occurs when the estimated differential current exceeds the set characteristic, as shown in Fig. 1^28^. The differential current low setting (*I*_*do*_) ranges from 0.20 to 2.50 *I*_*n*_, and the restraint factor (*K*_*s*_) lies between 0.10 and 1.0 (in steps of 0.01)^28^. The threshold value (*I*_*do*_) should be set above maximum load current to avoid tripping by the load current and CT errors, and it should be set at about 50% below minimum short-circuit currents to ensure tripping under minimum internal faults in power systems^28^. The change in the factor (*K*_*s*_) alters both tripping and stabilizing areas of the relay characteristic, as depicted in Fig. 1. A high setting for this factor can improve the stability with regard to faults outside the protection zone, but it can also reduce the sensitivity of fault detection. Therefore, the *K*_*s*_ factor should be selected as low as possible and as high as necessary. Moreover, a sensitive differential current monitoring with the parameter *I*_*dthr*_ can identify CT faults (such as CT short-circuits, open-circuits and reverse wiring even with load currents). The CT fault causes a differential current in the differential protection that will be then blocked, and an alarm will be declared. The setting of CT supervision differential current (*I*_*dthr*_) ranges from 0.1 *I*_*n*_ to 1.00 *I*_*n*_^28^.

The differential current (*i*_*ds*_*(k)*) at the ‘*k*’ sample for the ‘*S*’ phase of the protected busbar is calculated using the following formula:

1 $$i_{ds} (k) = \left| {\sum\limits_{m = 1}^{{m = N_{c} }} {i_{sm} (k)} } \right|$$

whereas, the restraining current (*i*_*rs*_*(k)*) at the ‘*k*’ sample for the ‘*S*’ phase of the protected busbar is computed as follows:

2 $$i_{rs} (k) = \sum\limits_{m = 1}^{{N_{c} }} {\left| {i_{sm} (k)} \right|}$$

It is obvious that *i*_*ds*_*(k)* and *i*_*rs*_*(k)* are estimated at every sample ‘*k*’ by processing the sequential samples. The amplitudes of the fundamental components of *i*_*ds*_*(k)* and *i*_*rs*_*(k)* are* I*_*ds*_ and *I*_*rs*_, respectively.

As shown in Fig. 1, the tripping characteristic of the relay during the first section ‘*AB*’ is determined by the following inequality^28^:

3 $$I_{ds} > I_{d0}$$

whereas, the tripping characteristic of the relay for the slope *(K*_*s*_) during the second section ‘*BC*’ is contingent on the following inequality^28^:

4 $$I_{ds} > I_{d0} + K_{s} \times I_{rs}$$

For normal load or through-fault currents, the differential current quantity is almost zero, while the restraint current quantity increases instantly. Whereas, both differential and restraint currents rise at the same time in the event of internal faults. Hence, the protection relay can decide whether there is an internal or external fault condition within a few milliseconds. Certain assumptions are made in order to achieve a proper relay operation, including that the type and ratio of CTs at both busbar ends are identical, that entering and exiting currents are exactly synchronized, and that CTs are dimensioned to ensure that all CTs transform currents without saturation for a duration of at least 4.0 ms^28^.

## Proposed method

### Basic concept

It is known that any fault current signal encompasses AC with/without DC components. The shape of the current signal is measured by the form factor, and the ripple content in the signal is measured by the ripple factor^36,37^. Both factors are dimensionless, and their quantities are typically expressed as a percentage. The ripple factor reveals the quality of the signal, which consists of AC and some DC parts, as well as its conversion efficiency to pure AC or pure DC components. The lower value of the ripple factor indicates a proper current signal, while the greater value of the ripple factor denotes a distortion in the current signal^36,37^. When the form factor is approaches unity and the ripple factor vanishes, both factors indicate the steady-state condition. Hence, a pure AC current signal is obtained (i.e., the signal is clear from the DC component content and without CT saturation extent). As a consequence, the ripple and form factors can be used to detect the CT saturation condition and the DC component of current signals. Furthermore, both factors are able to control a restraint factor/signal to prevent the differential relay operation during external faults with CT saturation presence. The characteristic slope of the differential protection can be adjusted online, and it can be modified based on the form and ripple factors of the current signals measured at the two terminals of the power system component. As a result, adaptive differential current relay characteristics based on the form and ripple factors of the busbar current signals can be established. The subsequent section will explain the numerical method for estimating the form and ripple factors.

#### Form and ripple factors

- For conversion to DC output:

The absolute value of the phase current waveform is regarded as a single-phase full-wave rectifier bridge. The ratio of the RMS value to the DC value of the current signal is defined as the Form Factor (*FF*_*i*_)^36,37^, which is a measure of the shape of the signal. The form factor (*FF*_*i*_) can be expressed as follows^36,37^:

5 $$FF_{i} = \frac{{I_{sRMS} }}{{I_{sDC} }}$$

The Ripple Factor (*RF*_*i*_) is another essential parameter used to measure the ripple content in the current signal^36,37^. Thus, it represents the smoothness of the current waveform. The *RF*_*i*_ is defined as the ratio between the effective RMS value of the AC component and the DC value of the measured current. The ripple factor (*RF*_*i*_) can be computed as follows^36,37^:

6 $$RF_{i} = \frac{{I_{sacRMS} }}{{I_{sDC} }} = \sqrt {\left( {\frac{{I_{sRMS} }}{{I_{sDC} }}} \right)^{2} - 1} = \sqrt {(FF_{i} )^{2} - 1}$$

The full-cycle RMS value (*I*_*sRMS*_) of the current signal can be calculated as follows^36,37^:

7 $$I_{sRMS} = \sqrt {\frac{1}{{N_{s} }}\sum\limits_{k = 1}^{{k = N_{s} }} {(i_{s} (k))^{2} } }$$

The full-cycle DC current (*I*_*sDC*_) is defined as the average value of the current signal per one cycle,^36,37^. The full-cycle DC component of the current signal can be quantified as follows:

8 $$I_{sDC} = \frac{1}{{N_{s} }}\sum\limits_{k = 1}^{{k = N_{s} }} {\left| {i_{s} (k)} \right|}$$

The proposed technique can apply the concept of moving data set, for calculating the form and ripple factors, whose size can be selected within the range of one cycle to a sub-cycle. The size of the data set can be configured as: 1/8, 1/4, 1/2, 3/4, or one cycle. In this study, the half-cycle period is selected to accelerate the relay operation. For this selection, the RMS and DC values (*I*_*sRMS*_ and *I*_*sDC*_) of the sinusoidal current signal can be calculated to obtain the form and ripple factors as described below.

The half-cycle RMS value of the current signal is quantified as follows^36,37^:

9 $$I_{sRMS} = \sqrt {\frac{1}{2\Pi }\int_{0}^{\Pi } {(I_{m} \sin (\omega t))^{2} \times d(\omega t)} } = \frac{{I_{m} }}{2}$$

The half-cycle DC value of the current signal is quantified as follows^36,37^:

10 $$I_{sDC} = \frac{1}{2\Pi }\int_{0}^{\Pi } {\left| {I_{m} \sin (\omega t) \times d(\omega t)} \right| = \frac{{I_{m} }}{\Pi }}$$

The half-cycle effective RMS value of the AC component for the current signal is calculated as follows^36,37^:

11 $$I_{sacRMS} = \sqrt {(I_{sRMS} )^{2} - (I_{sDC} )^{2} }$$

The half-cycle form factor (*FF*_*i*_) for the current signal is given by^36,37^:

12 $$FF_{i} = \frac{{I_{sRMS} }}{{I_{sDC} }} = \frac{\Pi }{2} = 1.57$$

The half-cycle ripple factor (*RF*_*i*_) for the current signal is given by^36,37^:

13 $$RF_{i} = \sqrt {(\frac{{I_{sRMS} }}{{I_{sDC} }})^{2} - 1} = \sqrt {(1.57)^{2} - 1} = 1.21$$

Table 1 lists the normal values of the form and ripple factors for ideal sinusoidal waveforms, in the instances of normal operation and steady-state fault current, at various data windows (including 1/8, 1/4, 1/2, 3/4, and one cycle).

**Table 1:** Form and ripple factors for ideal sinusoidal waveforms at different data window sizes.

Data window size	The estimated quantities					
	*I*_*sRMS*_	*I*_*sDC*_	*FF*_*i*_ = *I*_*sRMS*_* / I*_*sDC*_	*RF*_*i*_ = *I*_*sacRMS*_* / I*_*sDC*_	*FF*_*ix*_ = *I*_*sRMS*_* / I*_*sacRMS*_ *FF*_*ix*_ = *FF*_*i*_ × *RF*_*ix*_	*RF*_*ix*_ = *I*_*sDC*_* / I*_*sacRMS*_ *RF*_*ix*_ = *1/ RF*_*i*_
1 cycle	$I_{m} /\sqrt 2$	*2I*_*m*_*/π*	1.11	0.48	2.30	2.08
3/4 cycle	$I_{m} \sqrt 3 /(2\sqrt 2 )$	*3I*_*m*_*/2π*	1.28	0.80	1.60	1.25
1/2 cycle	$I_{m} /2$	***I***_***m***_***/****π*	**1.57**	**1.21**	**1.30**	**0.83**
1/4 cycle	$I_{m} /(2\sqrt 2 )$	*I*_*m*_*/2π*	2.22	1.98	1.12	0.51
1/8 cycle	$I_{m} \sqrt {\frac{\Pi /8 - 1/4}{{2\Pi }}}$	$(\frac{{I_{m} }}{2\Pi })(1 - \frac{1}{\sqrt 2 })$	3.23	3.07	1.05	0.33
Ideal values of *FF*_*i*_ = *1* Ideal values of *RF*_*i*_ = *0* Ideal values of *FF*_*ix*_ = *1* Ideal values of *RF*_*ix*_ = *0*						

Significant values are in bold.

(b) For conversion to AC output:

When converting to AC output, ripples may arise as a result of unfavorable DC components. This means that the AC signals should be pure sinusoidal waveforms (i.e., without any DC components) in the process of ideal conversion. Consider *RF*_*ix*_ to be the ratio between the effective value of the DC/ripple component and the RMS value of the AC component of the measured current. The mathematical formula for estimating the ripple factor (*RF*_*ix*_) is as follows:

14 $$RF_{ix} = \frac{{I_{sDC} }}{{I_{sacRMS} }} = \sqrt {\left( {\frac{{I_{sRMS} }}{{I_{sacRMS} }}} \right)^{2} - 1} = \sqrt {(FF_{ix} )^{2} - 1} = \frac{1}{{RF_{i} }}$$

Also, assume that *FF*_*ix*_ is the ratio between the RMS value and the RMS value of the AC component of the measured current. The mathematical equation for evaluating the form factor (*FF*_*ix*_) is as follows:

15 $$FF_{ix} = \frac{{I_{sRMS} }}{{I_{sacRMS} }} = FF_{i} \times RF_{ix}$$

When the data set size is a half-cycle, the values of *RF*_*ix*_ and *FF*_*ix*_ can be quantified using the following formula:

16 $$RF_{ix} = \frac{1}{{RF_{i} }} = \frac{1}{1.21} = 0.83$$

17 $$FF_{ix} = FF_{i} \times RF_{ix} = 1.57 \times 0.83 = 1.30$$

In fact, different unwanted ripples appear in power systems. Some of these ripples are inserted in the sinusoidal current signals due to DC component and CT saturation conditions. The CT saturation event causes loss of portions from the CT secondary current waveform, and this current loss flows in the CT iron core. Many applications of protection and control systems require that the ripple content doesn’t exceed a specified value, or the adaptation concept is used to avoid its negative effects on the action of these systems. Throughout this work, the developed adaptation procedure includes appropriate on-line modifying of the differential relay curves based on the estimated form and ripple factors, thus enabling improvement of protection stabilization for the situation of close external faults with high DC components.

### Adaptive differential relay scheme

The current data, measured at both ends of the protected equipment, are sufficient for processing the proposed algorithm utilizing the signals processing principal. The proposed algorithm identifies the fault condition, discriminates between internal and external faults, detects CT saturation, and estimates on-line the convenient slope for the differential relay characteristics. The more suitable slope of differential relay characteristic aims to make adaptive characteristics during CT saturation period to avoid false relay operation in the event of external faults. The adaptation process leads to extending the stabilizing area of the relay characteristic or accelerating the relay tripping during large internal fault currents with high DC components (i.e., relay operation is without pickup time delay). This is accomplished using the form and ripple factors calculated for each CT current signal. The strategy of the modified differential current protection can be described in the following sections:

#### Determination of fault inception instant (k_*f*_*)*

The proposed algorithm identifies the fault inception instant (*k*_*f*_) using the first derivative estimated for each CT current signal, and for each feeder connected to the protected busbar. In the majority of cases, the first derivative of the pre-fault current is negligible with respect to the first derivative of the post-fault current. Hence, the first derivative corresponding to each phase CT current can be considered a proper method for fault detection. In this method, if the first derivative has a value above a threshold value, a fault is ascertained; this is for at least one phase current signal. To determine the fault inception instant from the CT secondary current (*i*_*sm*_*(k)*) of each circuit ‘*m*’ and for each ‘*S*’ phase, the first derivative (*F*_*ism*_*(k)*) can be obtained as follows:

18 $${\text{F}}_{{{\text{ism}}}} (k) = \left| {\frac{{{\text{(i}}_{{{\text{sm}}}} {\text{(k)}}\begin{array}{*{20}c} {} \\ {} \\ \end{array} - \begin{array}{*{20}c} {} \\ {} \\ \end{array} {\text{i}}_{{{\text{sm}}}} {\text{(k - 1))}}}}{{\text{h}}}} \right|$$

where, *h* is the sampling time interval, and* f*_*s*_ is the sampling frequency (*h* = *1/f*_*s*_).

Then the proposed technique compares the estimated factor (*F*_*ism*_*(k)*) with a setting value (*F*_*s*_ = 200% *F*_*m*_) to determine the fault time inception (*k*_*f*_), where *F*_*m*_ is the maximum first derivative obtained from the stored pre-fault current. If the first derivative (*F*_*ism*_*(k)*) is greater than the setting value (*F*_*s*_), then the fault is identified at a sample index of *k*_*f*_ = *k*.

#### Fault directionality

The directionality factor (*DF*_*s*_), calculated using the input and output currents for each data window of each *‘S*’ phase of the protected equipment, can discriminate between the internal and external faults with/without CT saturation condition. The factor (*DF*_*s*_) is estimated instantly as follows:

19 $$DF_{s} (k) = \frac{{i_{\sin } (k) \times i_{sout} (k)}}{{\left| {i_{\sin } (k) \times i_{sout} (k)} \right|}}$$

where as, the factor (*DF*_*sv*_) can be formulated for each data window as follows:

20 $$DF_{sv} = \frac{1}{{N_{w} }} \times \left[ {\sum\limits_{k = 1}^{{k = N_{w} }} {\left( {\frac{{i_{\sin } (k) \times i_{sout} (k)}}{{\left| {i_{\sin } (k) \times i_{sout} (k)} \right|}}} \right)} } \right]$$

To discriminate between the cases of normal operation and external and internal faults with/without CT saturation presence, the proposed algorithm relies on the following rules:

(I) Normal operation and external fault without CT saturation conditions.
  If *DF*_*a*_ = *DF*_*b*_ = *DF*_*c*_ =  + 1 (i.e. the three directionality factors are + 1 for the three phases of the protected equipment), then this case is normal operating conditions or external faults without CT saturation extent. This condition results in *DF*_*av*_ = *DF*_*bv*_ = *DF*_*cv*_ =  + 1 during the periods of normal operation and external fault without CT saturation. Besides, the evaluated differential currents (*i*_*da*_*(k)*, *i*_*db*_*(k)* and *i*_*dc*_*(k)*) are below the set characteristic for the three phases of the protected equipment.
(II) Internal fault condition.
  If *DF*_*a*_* = − 1.0, DF*_*b*_ = − *1.0*, or *DF*_*c*_ =  − *1.0* (i.e., the directionality factor is ‘− 1.0’ for at least one phase of the protected equipment during the first data window after the fault time inception), then this case indicates to a fault located inside the protection zone. This state leads to *DF*_*av*_ < *0, DF*_*bv*_ < *0* or *DF*_*cv*_ < 0 during the period of internal fault. Additionally, the estimated differential current (*i*_*da*_*(k)*, *i*_*db*_*(k)* or *i*_*dc*_*(k)*) is above the set characteristic for at least one phase of the protected equipment. Hence, the faulted equipment must be isolated from the remaining power system.
(III) External fault with CT saturation condition.

In this situation, consider the time interval of the free saturated portion (after fault inception) for the distorted secondary current is at least one-eighths cycle (i.e., the minimum time-to-saturation is 1/8 cycle) because the CT secondary current does not saturate suddenly. Due to the case of external fault with CT saturation extent, the values of the three directionality factors (*DF*_*a*_*, DF*_*b*_, and *DF*_*c*_) or (*DF*_*av*_*, DF*_*bv*_, and *DF*_*cv*_) are positive values during the first data window after fault inception; besides the value of *DF*_*av*_*, DF*_*bv*_, or *DF*_*cv*_ isn’t equal to unity (i.e., the directionality factor is equal + 1.0 during the first window after the fault time inception for at least one phase of the protected equipment). In other words, the value of directionality factor for at least one phase isn’t equal to + 1.0, and the values of the other directionality factors of the remaining phases are equal to + 1.0. This case results in + 1.0 > *DF*_*av*_ ≥ *0.0,* + 1.0 > *DF*_*bv*_ ≥ *0.0* or + 1.0 > *DF*_*cv*_ ≥ 0.0 during the time snap of external fault with CT saturation condition. Moreover, the calculated differential currents (*i*_*da*_*(k)*, *i*_*db*_*(k)*, and *i*_*dc*_*(k)*) are below the set characteristic for the three phases during the free saturated portions of the secondary currents, while they are above the set characteristic for at least one phase during the saturated portions of secondary currents. Consequently, this event makes the protection scheme implements the adaptation process for relay operating characteristics. The values range of the three-phase directionality factors and differential currents for each fault type is given in Table 2. As shown in the table, it is obvious that the values of directionality factors are restricted between − 1.0 and + 1.0, while the magnitudes of differential currents aren’t limited.

**Table 2:** The values range of three-phase directionality factors and differential currents at different fault types and the traditional relay action.

Sr. No.	Fault type	The range values of the three phase directionality factors	The range values of the three phase differential currents	Traditional relay action
1.	Normal operation	*DF*_*av*_ = + 1.0 *DF*_*bv*_ = + 1.0 *DF*_*cv*_ = + 1.0	*i*_*da*_ < *I*_*d0*_ + *K*_*s*_ × *i*_*ra*_, *i*_*db*_ < *I*_*d0*_ + *K*_*s*_ × *i*_*rb*_ and *i*_*dc*_ < *I*_*d0*_ + *K*_*s*_ × *i*_*rc*_	Blocking
2.	External fault without CT saturation condition	*DF*_*av*_ = + 1.0 *DF*_*bv*_ = + 1.0 *DF*_*cv*_ = + 1.0	*i*_*da*_ < *I*_*d0*_ + *K*_*s*_ × *i*_*ra*_, *i*_*db*_ < *I*_*d0*_ + *K*_*s*_ × *i*_*rb*_ and *i*_*dc*_ < *I*_*d0*_ + *K*_*s*_ × *i*_*rc*_	Blocking
3.	External fault with CT saturation condition	+ 1.0 > *DF*_*av*_ ≥ *0.0,* + 1.0 > *DF*_*bv*_ ≥ *0.0,* or + 1.0 > *DF*_*cv*_ ≥ 0.0	*i*_*da*_ < *I*_*d0*_ + *K*_*s*_ × *i*_*ra*_, *i*_*db*_ < *I*_*d0*_ + *K*_*s*_ × *i*_*rb*_ and *i*_*dc*_ < *I*_*d0*_ + *K*_*s*_ × *i*_*rc*_ during the free saturated portions of the secondary currents	The CT saturation may lead to the traditional relay malfunction (Tripping) on severe external faults
			*i*_*da*_ ≥ *I*_*d0*_ + *K*_*s*_ × *i*_*ra*_, *i*_*db*_ ≥ *I*_*d0*_ + *K*_*s*_ × *i*_*rb*_ or *i*_*dc*_ ≥ *I*_*d0*_ + *K*_*s*_ × *i*_*rc*_ during the saturated/distorted portions of secondary currents	
4.	Internal fault without CT saturation condition	0.0 > *DF*_*av*_ ≥ *-1.0,* *0.0* > *DF*_*bv*_ ≥ *-1.0,* or *0.0* > *DF*_*cv*_ ≥ -1.0	*i*_*da*_ ≥ *I*_*d0*_ + *K*_*s*_ × *i*_*ra*_, *i*_*db*_ ≥ *I*_*d0*_ + *K*_*s*_ × *i*_*rb*_ or *i*_*dc*_ ≥ *I*_*d0*_ + *K*_*s*_ × *i*_*rc*_	Tripping
5.	Internal fault with CT saturation condition	0.0 > *DF*_*av*_ ≥ *-1.0,* *0.0* > *DF*_*bv*_ ≥ *-1.0,* or *0.0* > *DF*_*cv*_ ≥ -1.0	*i*_*da*_ ≥ *I*_*d0*_ + *K*_*s*_ × *i*_*ra*_, *i*_*db*_ ≥ *I*_*d0*_ + *K*_*s*_ × *i*_*rb*_ or *i*_*dc*_ ≥ *I*_*d0*_ + *K*_*s*_ × *i*_*rc*_ during the free saturated portions of the secondary currents	The presence of harmonics (the DC components, CT saturation, or both together) may delay or prevent the traditional relay operation on severe internal faults
		+ 1.0 > *DF*_*av*_ ≥ *0.0,* + 1.0 > *DF*_*bv*_ ≥ *0.0,* or + 1.0 > *DF*_*cv*_ ≥ 0.0	*i*_*da*_ < *I*_*d0*_ + *K*_*s*_ × *i*_*ra*_, *i*_*db*_ < *I*_*d0*_ + *K*_*s*_ × *i*_*rb*_ or *i*_*dc*_ < *I*_*d0*_ + *K*_*s*_ × *i*_*rc*_ during the saturated/distorted portions of secondary currents	

#### CT saturation detection

The proposed algorithm performs three criteria sequentially processed (during the first window after the fault time inception) to affirm the CT saturation condition as follows:

(I) The estimated three-phase directionality factors are used to detect the CT saturation state based on the rules mentioned in the previous section.
(II) The calculated form and ripple factors of each individual CT secondary current can be used to identify the CT saturation condition. This is based on the following concept:
  In the cases of normal operation, no CT saturation and no DC component extent in fault currents, the factor (*RF*_*i*_) is fixed and close to the value of 1.21 (i.e., *RF*_*ix*_ = 0.83). This means that the form and ripple factors (*FF*_*i*_*, RF*_*i*_*, FF*_*ix*_ and *RF*_*ix*_) are normal values as given in Table 1. Whereas in the cases of CT saturation extent and DC component content in the fault currents, the factor (*RF*_*i*_) is not constant, and it becomes greater than a selected threshold value of )*1.21* + *Δ*(= *1.31*. Also, the factor (*RF*_*ix*_) is not constant, and it becomes lower than the selected threshold value of 0.76 (i.e., 1/1.31 = 0.76). The values of the two factors (*FF*_*i*_ and *RF*_*i*_) change according to the CT saturation level and the asymmetrical component extent. The more CT saturation severity level, the more values of form and ripple factors (*FF*_*i*_ and *RF*_*i*_) and also the lower values of the two factors (*FF*_*ix*_ and *RF*_*ix*_).
(III) The differential current (*i*_*ds*_*(k)*) is useful to differentiate between internal fault and external fault with CT saturation. If the magnitude of the differential current quantity, during the interval following the fault time inception and before the CT saturates for at least one-eighths cycle, is lower than a predefined threshold value, a fault location is outside the equipment protection zone; otherwise, the fault is internal. The external fault with CT saturation extent is defined from the calculated differential current (*i*_*ds*_*(k)*), which is above the set characteristic after a delay time (*T*_*d*_) from the fault time starting. After the delay time (*T*_*d*_), the differential current (*i*_*ds*_*(k)*) transfers between the tripping and blocking areas of the relay characteristic.

#### Calculation of adaptive characteristic slope

The protection technique is based on estimating the suitable slope for the relay characteristics, using the form and ripple factors (*FF*_*i*_ and *RF*_*i*_) or (*FF*_*ix*_ and *RF*_*ix*_) calculated for the current signals, in order to produce a restraint signal that prevents the relay operation during external faults with CT saturation presence. In this instance, the stabilizing area must be increased with the increase in the CT saturation level. The greater the form and ripple factors* (FF*_*i*_ and *RF*_*i*_*),* the greater the operating characteristic slope (*K*_*s*_) of the differential relay*.* As a result, the relation between the characteristic slope (*K*_*s*_) and the two factors* (FF*_*i*_ and *RF*_*i*_*)* is proportional linear. The convenient slope is estimated for each data window of half-cycle. For estimating the operating characteristic slope (*K*_*s*_), the two factors* (FF*_*ix*_ and *RF*_*ix*_*)* are used*.*

In the ideal case, *I*_*sDC*_ = *0* and *I*_*sRMS*_ = *I*_*sacRMS*_. As a result, *FF*_*ix*_ =  + *1* and *RF*_*ix*_ = *0*. Whereas, in the presence of the CT saturation and the DC components of the fault current, *I*_*sDC*_* > 0* and *I*_*sRMS*_*  > I*_*sacRMS*_. As a consequence, *FF*_*ix*_*  >* + *1* and *RF*_*ix *_* > 0.*

In this scenario, consider that the selected data window is half-cycle and the differential relay characteristic with slope *K*_*s*_ varies from 0.1 to 1.0 like Siemens manufacturer^28^. Initially, select the slope *K*_*s1*_ = 0.0 when *FF*_*i*_ = 1.0 (for the ideal case) and *K*_*s1*_ = 1 when *FF*_*i*_ = 2.3 (for the worst case in Table 1). Also, select the slope *K*_*s2*_ = 0.0 when *RF*_*i*_ = 0.0 (for the ideal case) and *K*_*s2*_ = 1 when *RF*_*i*_ = 2.08 (for the worst case in Table 1). By substituting these values into both two linear Eqs. (21) and (25), the values of the constants *c*_*1*_, *c*_*2*_, *c*_*3*_ and *c*_*4*_ are 0.77, − 0.77, 0.48 and 0.0, respectively. The adaptive tripping characteristics slopes (*K*_*s1*_ and* K*_*s2*_) can be calculated by Eqs. (24) and (28).

21 $$K_{s1} = c_{1} \times FF_{ix} + c_{2}$$

22 $$0.0 = c_{1} \times 1.0 + c_{2}$$

23 $$1 = c_{1} \times 2.3 + c_{2}$$

24 $$K_{s1} = 0.77 \times FF_{ix} - 0.77$$

25 $$K_{s2} = c_{3} \times RF_{ix} + c_{4}$$

26 $$0.0 = c_{3} \times 0.0 + c_{4}$$

27 $$1 = c_{3} \times 2.08 + c_{4}$$

28 $$K_{s2} = 0.48 \times RF_{ix}$$

From the tripping characteristics slopes (*K*_*s1*_ and* K*_*s2*_), it is often more convenient to select:

- The tripping characteristic slopes *K*_*s1*_ = *K*_*s2*_ = *0.0* for the ideal case without ripples content, where *FF*_*ix*_ =  + 1 and *RF*_*ix*_ = 0*,* respectively,
- The tripping characteristic slopes *K*_*s1*_ = *0.23* and* K*_*s2*_ = *0.4* are suitable for normal operation with ripple content and for no CT saturation extent, due to the linear CTs errors, where *FF*_*ix*_ = *1.30* and *RF*_*ix*_ = *0.83,* respectively, and.
- The operating characteristic slopes *K*_*s1*_ = *K*_*s2*_ =  + 1 are suitable for the worst degree of CT saturation where *FF*_*ix*_ = *2.30* and *RF*_*ix*_ = *2.08,* respectively, due to the linear and non-linear CT errors.

**Fig. 2:**
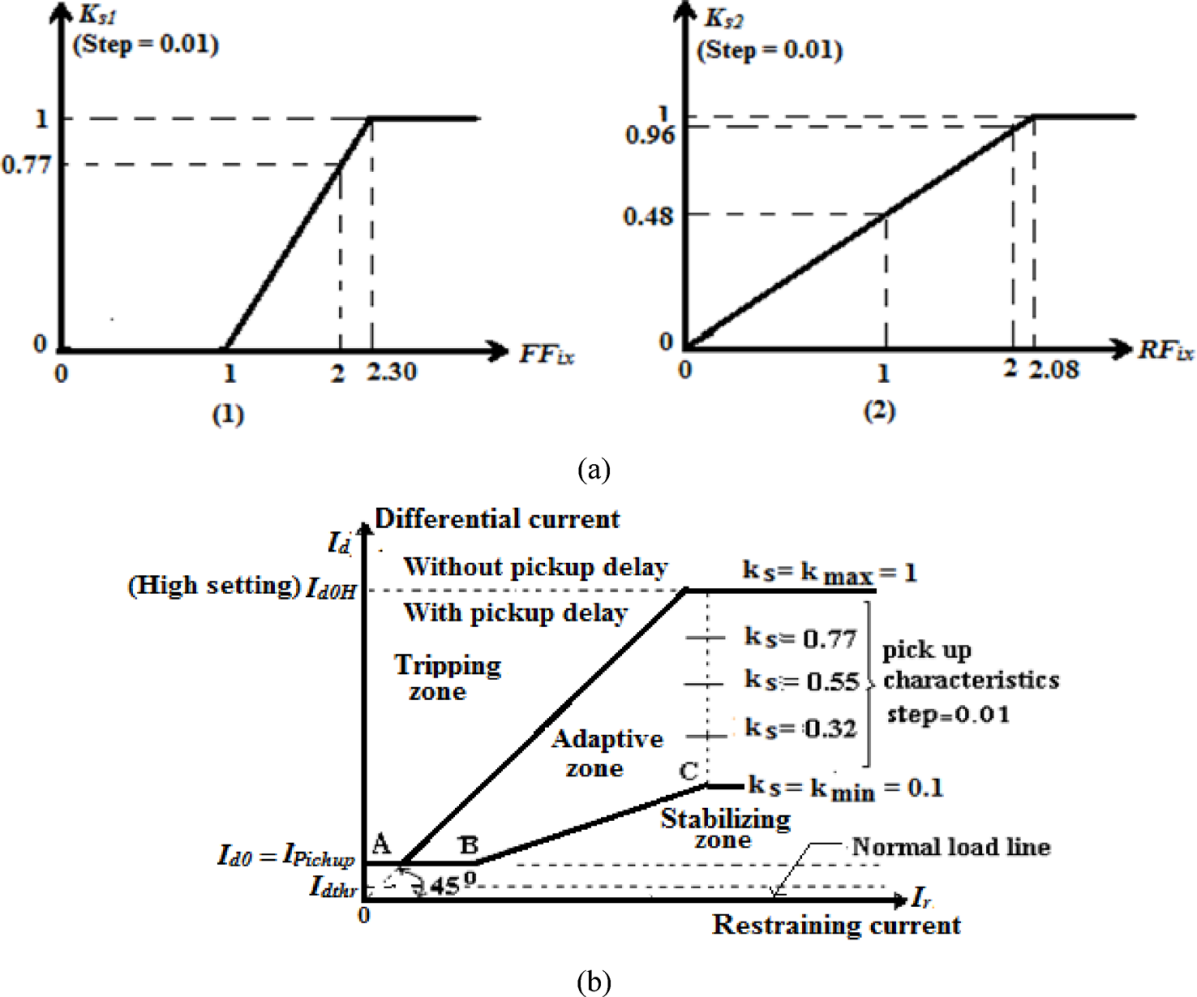

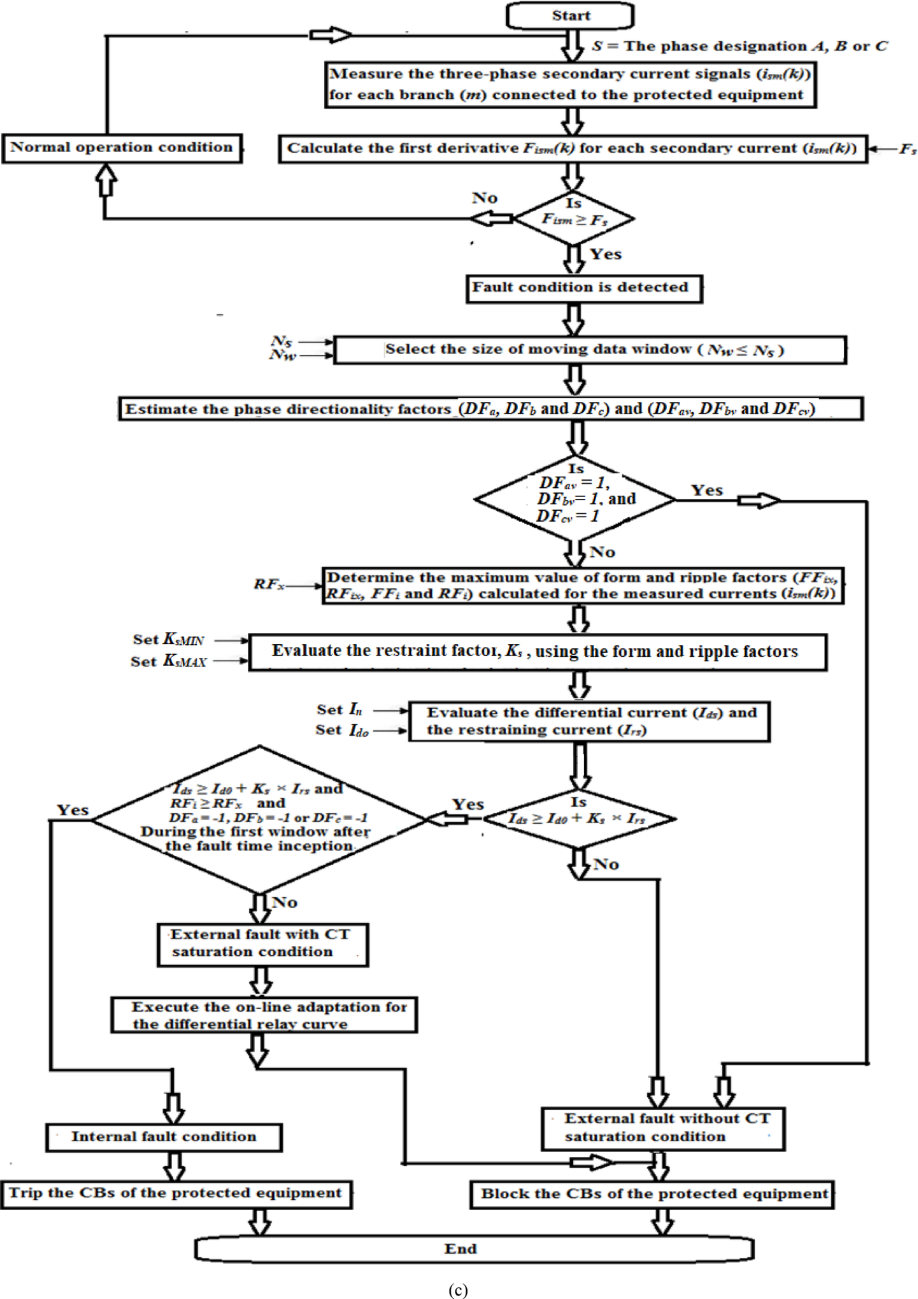
(**a**) The relation between the two tripping characteristics slopes (*K*_*s1*_ and* K*_*s2*_) and the two factors (*FF*_*ix*_ and *RF*_*ix*_), respectively. (**b**) Adaptive characteristics of the biased differential relay. (**c**) Flow chart of the adaptive differential protection scheme based on the form and ripple factors for the current signals.

Figure 2a illustrates the relation between the two tripping characteristics slopes (*K*_*s1*_ and* K*_*s2*_*)* and the two factors (*FF*_*ix*_ and *RF*_*ix*_), respectively. Now, the online adaptation process can be applied by selecting only one formula of the two mathematical formulas (*K*_*s1*_ and* K*_*s2*_*)* for calculating the suitable operating characteristic slope.

In this study, the form and ripple factors (*FF*_*ix*_ and *RF*_*ix*_) can be used to:

1. Figure out and assess the intensity level of the CT saturation,
2. Estimate the DC component percentage in the fault current, and.
3. Determine the curve slope for the adaptive differential relay characteristic.

#### Adaptive operating characteristics

Figure 2b shows the adaptive characteristics of the biased differential relay. The novel adaptive tripping characteristics based on the form and ripple factors estimated for the measured currents represent a development for the conventional differential current relays. Each operating characteristic includes three regions as follows:

(I) The tripping zone, which can be used to detect internal fault events, then the faulted equipment must be isolated from the remaining power system. Consequently, the proposed scheme action sets the trip signal to a high value of (1),
(II) The blocking zone, which can be used to prevent isolating/tripping equipment CBs in the case of external faults or normal operating instances. As a consequence, its action holds the trip signal to a low value of (0), and
(III) The adaptive zone, which can be used to adjust the tripping characteristic of the differential current relay in response to CT saturation extent or DC component content of fault currents. This is accomplished by estimating the suitable slope for the relay characteristic using the form and ripple factors calculated for the measured current signals to produce a convenient restraint factor/signal. The relay operation during the external faults with CT saturation can be inhibited. Moreover, it performs other protection functions to differentiate between the instances of internal and external faults.

#### Adaptive algorithm procedure

Figure 2c presents the flow chart of the adaptive differential protection scheme based on the form and ripple factors for the measured current signals measured at the input and output terminals of the protected power element. The proposed algorithm of adaptive differential protection characteristics can be sequentially processed as follows:

1. Measure the data of each ‘*S*’ phase secondary current signal (*i*_*sm*_*(k)*) for each branch (*m*) connected to the protected equipment,
2. Select the setting value (*F*_*s*_) of the first derivative,
3. Calculate the first derivative (*F*_*ism*_*(k)*) for each secondary current (*i*_*sm*_*(k)*) measured from each phase CT. This is to identify the fault instant *(k*_*f*_*)*; a transition is detected for each phase and for each feeder connected to the equipment,
4. Set the number of samples per cycle and the number of samples per moving data window (*N*_*s*_ and *N*_*w*_*,* respectively),
5. Estimate the phase directionality factors (*DF*_*a*_*, DF*_*b*_, and *DF*_*c*_) and (*DF*_*av*_*, DF*_*bv*_, and *DF*_*cv*_),
6. Define the event of external faults without CT saturation using the phase directionality factors (*DF*_*av*_*, DF*_*bv*_, and *DF*_*cv*_),
7. Select the ripple factor limit (*RF*_*x*_),
8. Estimate the maximum values of form and ripple factors (*FF*_*ix*_*, RF*_*ix*_*, FF*_*i*_ and *RF*_*i*_) for all CTs secondary current signals (*i*_*sm*_*(k)*),
9. Set the equipment nominal current, pickup differential current, and the minimum and maximum slopes of the characteristic curve (*I*_*n*_*, I*_*do*_, *K*_*sMIN*_*,* and *K*_*sMAX*_*,* respectively),
10. Evaluate the new tripping characteristic slope (*K*_*s*_ = *K*_*s1*_ or *K*_*s*_ = *K*_*s2*_) using the maximum values of the form and ripple factors (*FF*_*ix*_ and *RF*_*ix*_), which is able to modify the ratio between the stabilizing and tripping zones within the differential relay characteristic curve,
11. Calculate the vector sum of the differential current (*i*_*ds*_*(k)*) and the algebraic sum of the restraining current (*i*_*rs*_*(k)*) using the measured secondary currents for each phase of the protected equipment,
12. Compare between the differential and restraining currents (*i*_*ds*_ and *I*_*d0*_ + *K*_*s*_ × *i*_*rs*_) for each phase,
13. Differentiate between the instances of internal and external faults with CT saturation extent, and
14. A relay response is as follows:
  (i) In the instance of internal faults: Trip the CBs of the protected equipment,
  (ii) In the instance of external faults without CT saturation condition: Block the CBs of the protected equipment,
  (iii) In the instance of external faults with CT saturation condition: execute the online adaptation process of the differential relay curve during the periods of CT saturation and DC component content of the fault current.

## Power system description

**Fig. 3:**
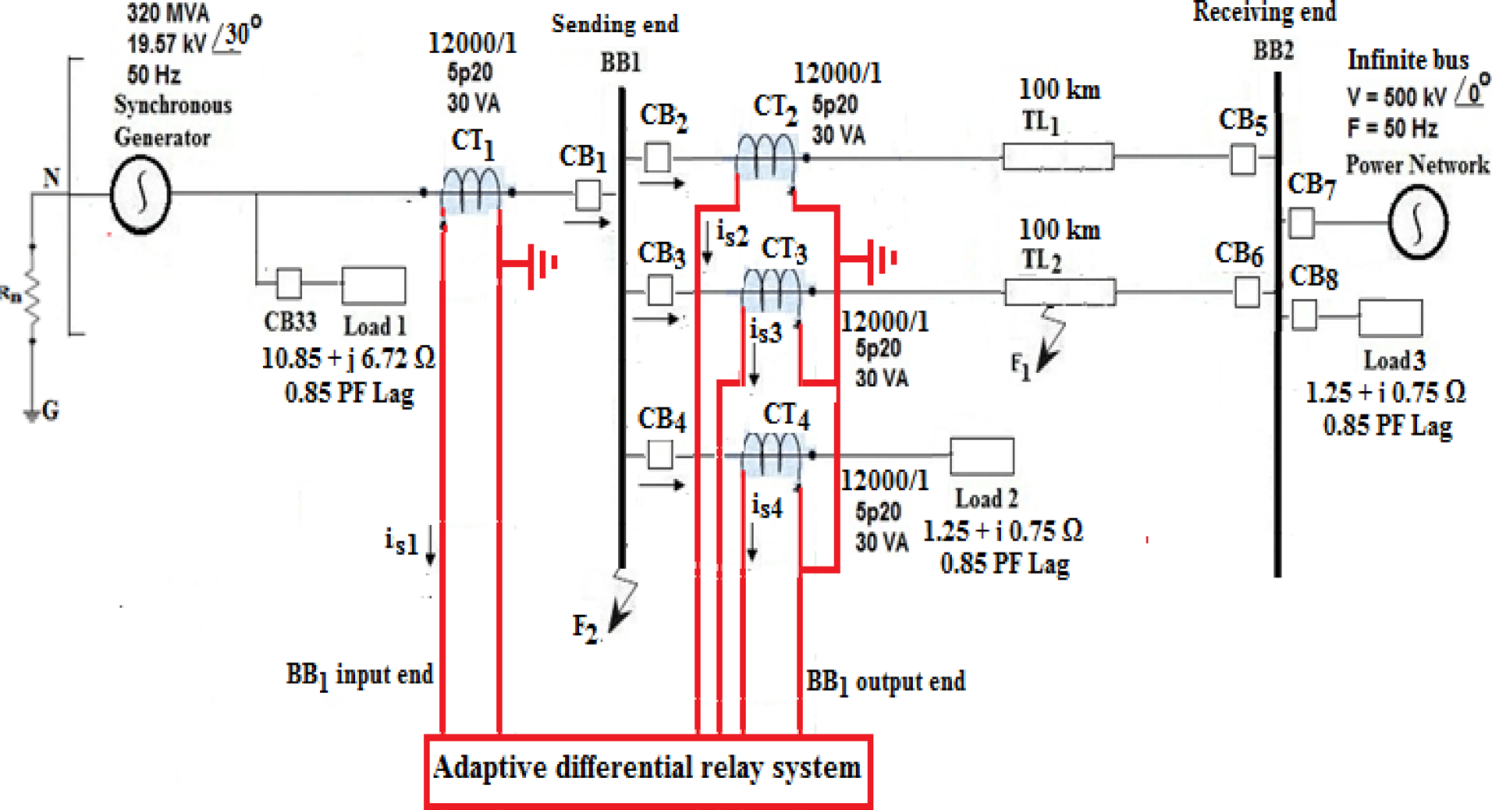
SLD of the simulated power system.

The *SLD* of the power system model under study is shown in Fig. 3, which is used to verify the developed scheme. The model is a segment of a medium-voltage power system. It is composed of double-circuits of 19.57 kV; each *TL* has a length of 100 km and extends between a synchronous machine and a connected electrical network via two busbars (*BB*_*1*_ and *BB*_*2*_), besides three electrical loads. Each *TL* is represented by distributed parameters, and the frequency dependence of the line parameters is taken into account. The components of the system model, simulated by the Alternative Transient Program (*ATP*), are based on realistic parameters given in Appendix 1^38^. To validate the proposed technique, extensive simulation case studies have been carried out under variable operating and fault conditions using the *ATP* software, and the algorithm operation has been implemented using the *MATLAB/M-file* environment.

## Simulations and results analysis

In the proposed approach, the data of three-phase currents are taken from the four circuits connected to the first busbar (*BB*_*1*_). The data is used to evaluate the first derivative, the directionality factors, and the form and ripple factors for each current signal, the adaptive slope, and the differential and restraint currents for each phase to perform the adaptation process. This work aims to adjust the differential relay characteristics in the case of changes in CT saturation level and the DC component content of fault currents (to enhance the relay stability and security). In this study, the CT distorted currents and DC components arise from both internal and external faults. Moreover, some assumptions are required in order to get a proper relay operation as follows: (1) identical CT type on both ends of the protected equipment, (2) equal CT ratio, (3) currents at both *BB*_*1*_ ends are exactly synchronized, and (4) CTs are dimensioned such that all CTs transform currents without saturation for a time at least equals 2.5 ms. The *BB*_*1*_ currents are generated using the *ATP* program with a sampling time of 0.2 ms, and the total simulation time is 0.3 s (i.e., the total number of samples is *N*_*sim*_ = 1500 samples). Appendix 2 contains the input data of the proposed protection algorithm.

Numerous simulation situations have been tested under various operating and fault conditions. The performance of the algorithm is evaluated under the impact of variations of fault and power system parameters such as fault type (*SLG, DLG, DL* and *3LG*), fault resistance (0–200 Ω), fault inception angle (0–360 °), fault location (internal/external), CT burden (0–500 Ω), CT ratio (12,000/1–1000/1) and power flow angle. For each case study, consider the external faults are situated at the point *F*_*1*_ located on 25% of *TL*_*2*_ length starting from the relay place (installed at busbar *BB*_*1*_*)*, and the internal faults are located at the point *F*_*2*_ located on the busbar *BB*_*1*_. Also, assume that the power system is loaded before the fault inception. The pre-fault operating conditions of the simulated power system are included in Appendix 1. For each case, *δ*_*1*_= *30°*, *δ*_*2*_ = *0°*, *F*_*1Operated*_ = *F*_*2Operated*_ = *50 Hz*, and* V*_*1Max*_ = *V*_*2Max*_ = *16.063 kV.*

### Case study 1: external SLG fault with CT saturation

**Fig. 4:**
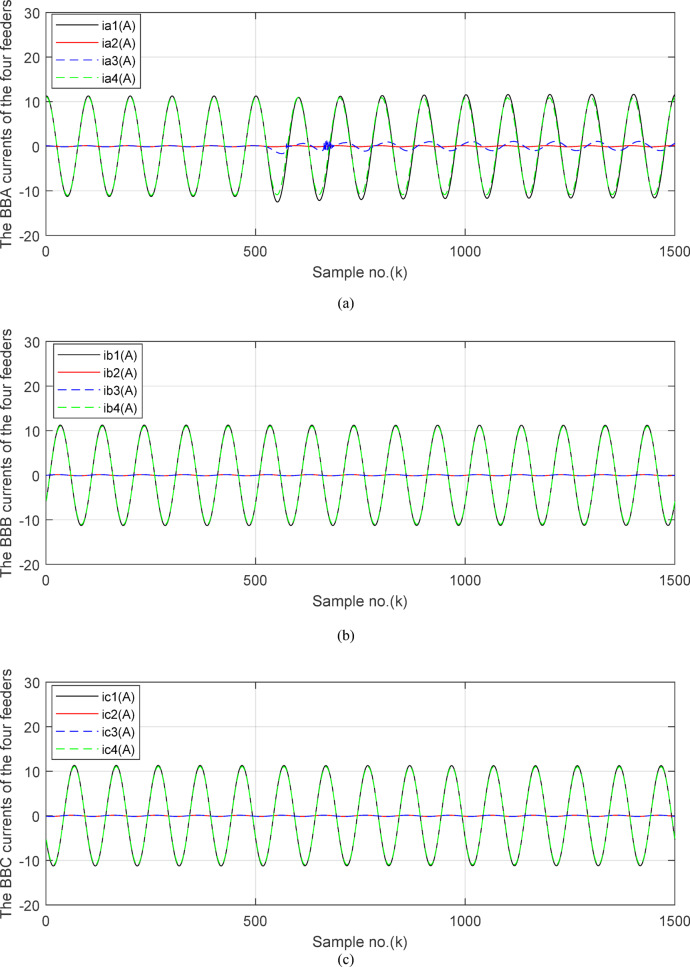

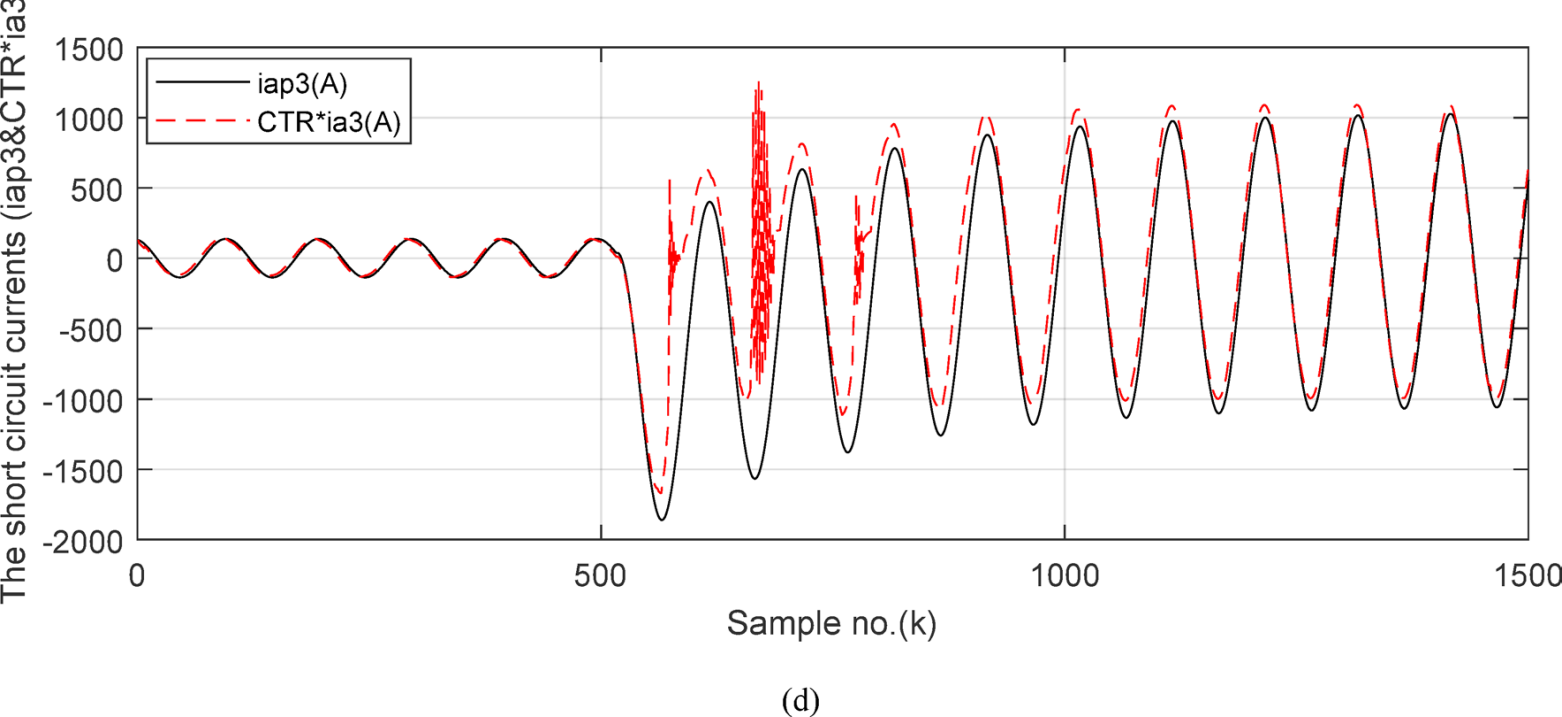
(**a**) The ‘*A*’ phase secondary currents of the four feeders. (**b**) The ‘*B*’ phase secondary currents of the four feeders. (**c**) The ‘C’ phase secondary currents of the four feeders. (**d**) The ‘A’ phase primary and secondary (referred to primary side) currents of the third feeder (TL2 = the faulted feeder). (**a**-**d**) Simulation current signals for case study 1.

**Fig. 5:**
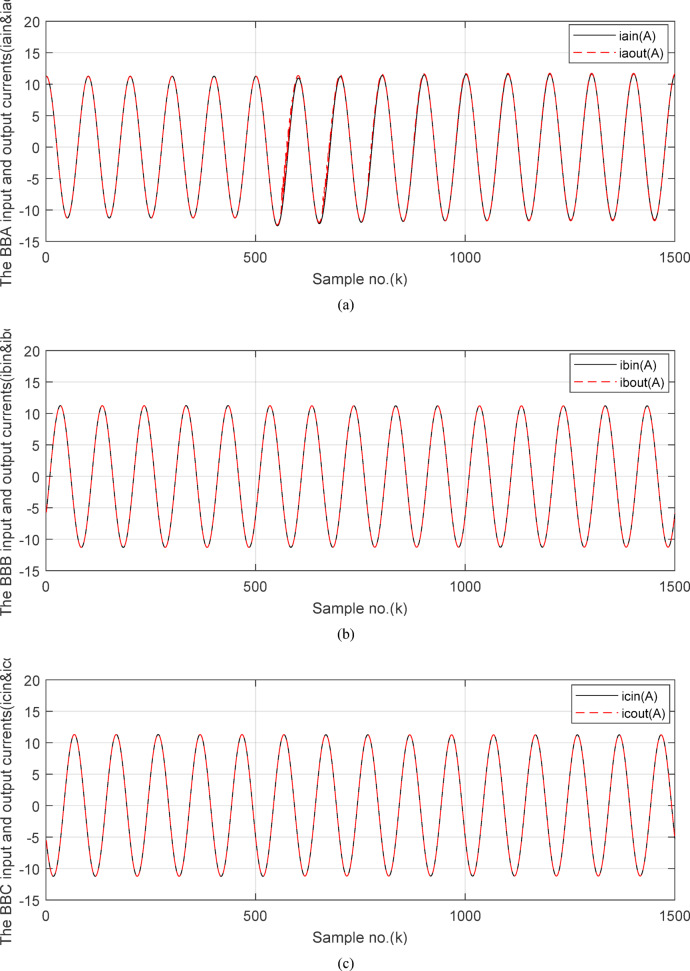

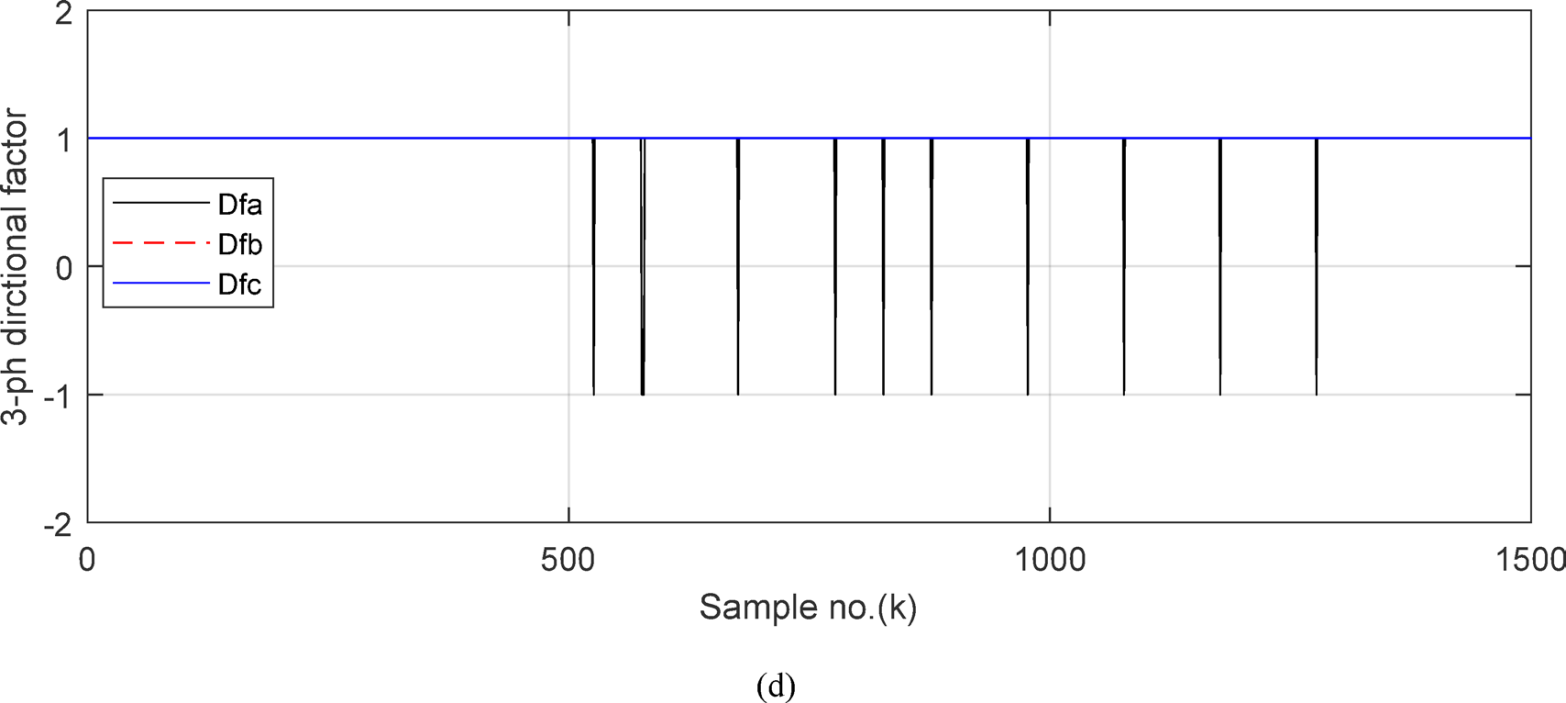
(**a**) The input and output secondary current signals (*i*_*ain*_ and *i*_*aout*_) for the ‘*A*’ phase of *BB*_*1*_. (**b**) The input and output secondary current signals (*i*_*bin*_ and *i*_*bout*_) for the ‘*B*’ phase of *BB*_*1*_. (**c**) The input and output secondary current signals (*i*_*cin*_ and *i*_*cout*_) for the *‘C*’ phase of *BB*_*1*_. (**d**) The instantaneous values of three-phase directionality factors (*DF*_*a*_*, DF*_*b*_ and *DF*_*c*_) in the case of external fault with CT saturation*.* (**a–d**) Simulation current signals and the directionality factors for case study1.

**Fig. 6:**
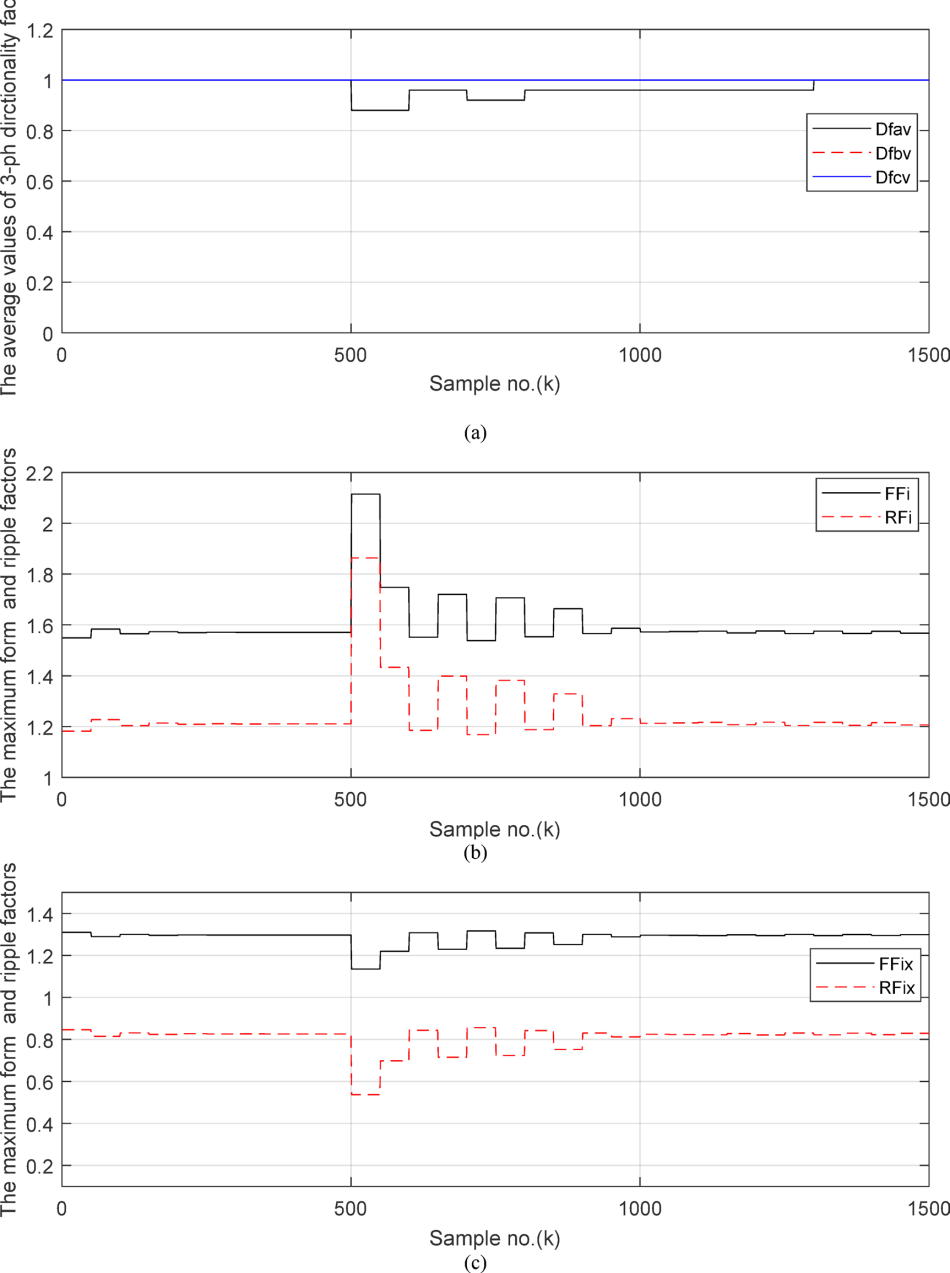

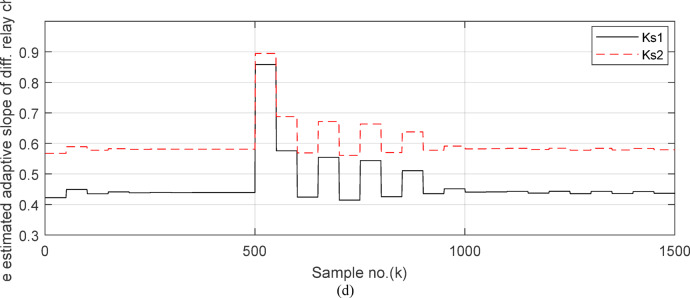
(**a**) The average values of three phases directionality factors (*DF*_*av*_*, DF*_*bv*_ and *DF*_*cv*_) in the case of external fault with CT saturation. (**b**) The calculated maximum form and ripple factors (*FF*_*i*_ and *RF*_*i*_). (**c**) The calculated form and ripple factors (*FF*_*ix*_ and *RF*_*ix*_). (**d**) The calculated operating characteristic slope of differential relay (*K*_*s1*_ and *K*_*s2*_). (**a–d**). Response of adaptive busbar differential relay for case study 1.

**Fig. 7:**
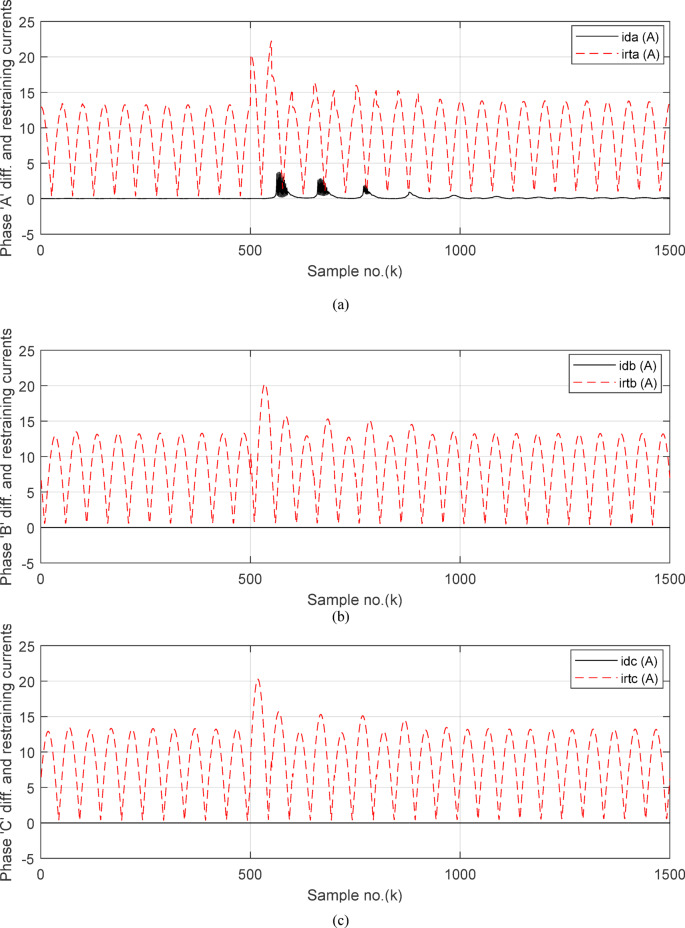
(**a**) The instantaneous differential and restraining current signals (*i*_*da*_ and *I*_*d0*_ + *K*_*s2*_ × *i*_*ra*_) for the ‘*A*’ phase of *BB*_*1*_. (**b**) The instantaneous differential and restraining current signals (*i*_*db*_ and *I*_*d0*_ + *K*_*s2*_ × *i*_*rb*_) for the ‘*B*’ phase of *BB*_*1*_. (**c**) The instantaneous differential and restraining current signals (*i*_*dc*_ and *I*_*d0*_ + *K*_*s2*_ × *i*_*rc*_) for the ‘*C*’ phase of *BB*_*1*_. (**a–c**) The calculated instantaneous differential and restraining current signals for case study 1.

**Fig. 8:**
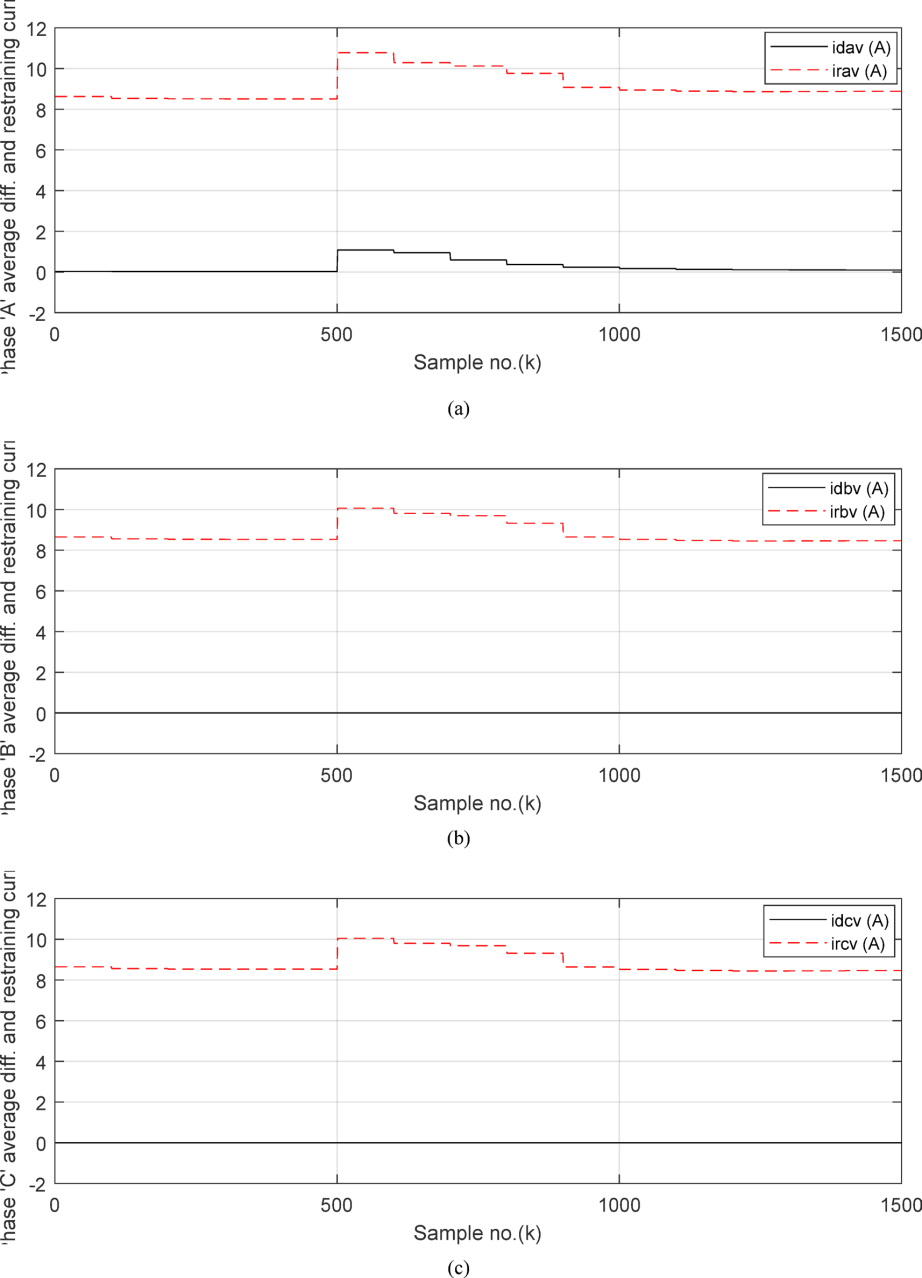

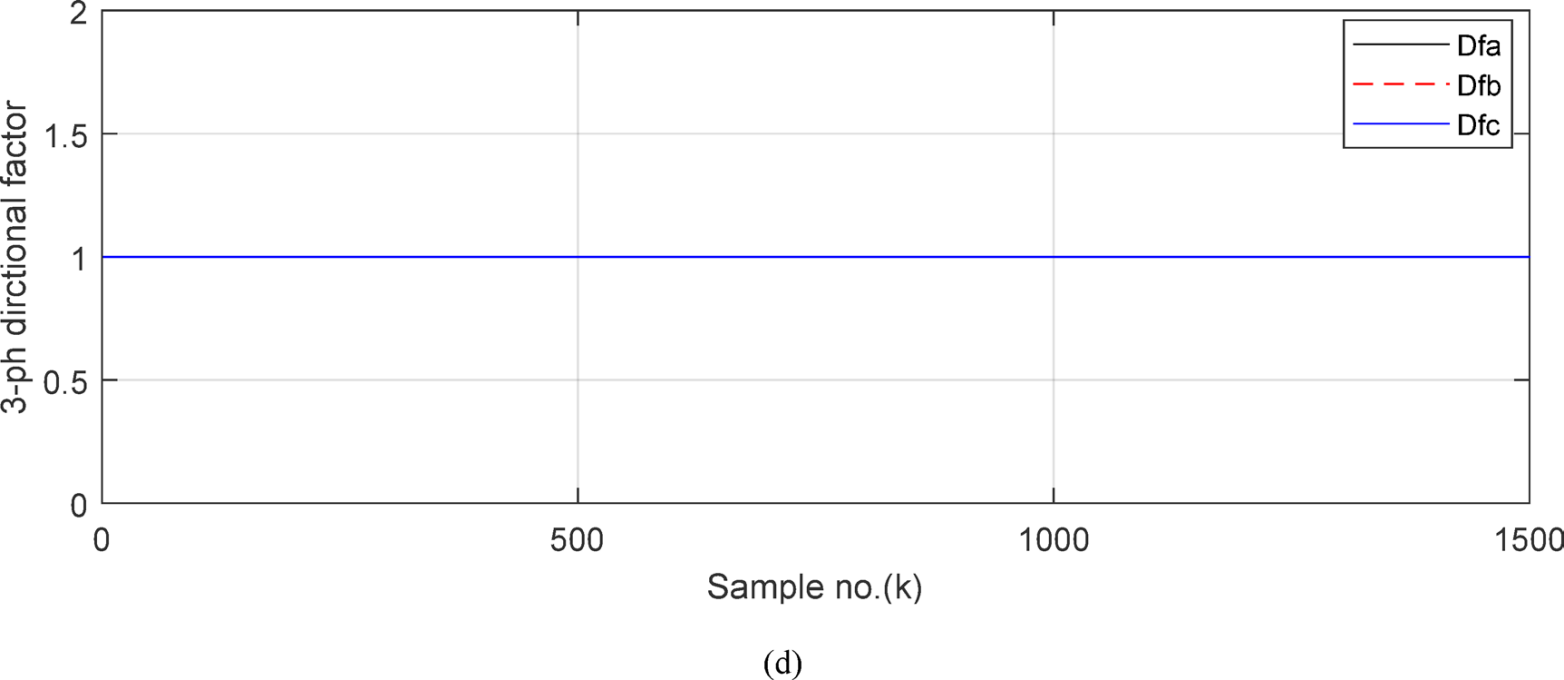
(**a**) The average value of differential and restraining current signals (*i*_*da*_ and *I*_*d0*_ + *K*_*s2*_ × *i*_*ra*_) for the ‘*A*’ phase of *BB*_*1*_. (**b**) The average value of differential and restraining current signals (*i*_*db*_ and *I*_*d0*_ + *K*_*s2*_ × *i*_*rb*_) for the ‘*B*’ phase of *BB*_*1*_. (**c**) The average value of differential and restraining current signals (*i*_*dc*_ and *I*_*d0*_ + *K*_*s2*_ × *i*_*rc*_) for the ‘*C*’ phase of *BB*_*1*_. (**d**)The three-phase directionality factors (*DF*_*a*_*, DF*_*b*_ and *DF*_*c*_) in the cases of normal operation and external fault without CT saturation and *R*_*b*_ = 0.5 Ω. (**a–c**) The calculated average differential and restraining current signals for case study 1.

In this case study, the simulation is carried out to evaluate the performance of the proposed technique in the instance of the external fault with CT saturation extent. An external *SLG* fault without fault resistance (*R*_*f*_ = 0 *Ω*) is simulated at the point *F*_*1*_ located at 25% of *TL*_*2*_ length for the ‘*A*’ phase. For this case, the fault inception time is *t*_*f*_ = 0.103 S. This means that the sample index where the fault time inception is *k*_*f*_ = 515. The CTR is changed from 12,000/1 to 1000/1 for each CT of all feeders connected to the *BB*_*1*_. The CT burden for all feeders is *R*_*b*_ = 0.5 + j0.0 Ω except the CT burden *R*_*b*_ = *500 Ω* for *‘A*’ phase CT of the third branch (*TL*_*2*_). Figures 4, 5, 6, 7 and 8 show the simulation results for case study 1. Figures 4a–c present the three-phase secondary currents of the four circuits (including generator, *TL*_*1*_*, TL*_*2*_ and Load ‘2’ circuits) connected to the protected busbar (*BB*_*1*_), respectively, in the case of the external *SLG* fault with CT saturation. Figure 4d plots the primary and saturated secondary currents (referred to as the primary side) for the ‘*A*’ phase of the branch of *TL*_*2*_ (i.e., the faulted and third circuit). It is noticed that for the ‘*A*’ phase of the faulted circuit, the current signal during the fault time interval is higher than the pre-fault current; the fault current is greater than 6 × *I*_*n*_, and the current signal is with a large asymmetrical component. It is obvious that the high magnitude of short-circuit current, the low CTR, the large CT burden, and the asymmetry in the current waveform during the transient period are attributed to CT saturation for the ‘*A*’ phase current of the faulted circuit (*TL*_*2*_). Also, it is seen that the above-mentioned reasons result in low time-to-saturation, where the CT saturation condition reveals during the first half-cycle of the fault time.

Figures 5a–c show the calculated input and output current signals of the *A, B* and *C* phases for the protected busbar (*BB*_*1*_), respectively. As illustrated in Figs. 5b–c, the input and output current signals of ‘*B*’ and ‘*C*’ phases are in phase and identical before and during the fault span. Whereas, the input and output current signals for the ‘*A*’ phase of *BB*_*1*_ are symmetrical before the fault time, while their values are slightly out of phase during the first four cycles of the fault time. This is a result of the CT saturation extent, as depicted in Fig. 5a. Figure 5d offers the instantaneous values of the three-phase directionality factors (*DF*_*a*_*, DF*_*b*_ and *DF*_*c*_) in the case of the external fault with CT saturation for the ‘*A*’ phase of the faulted circuit (*TL*_*2*_). As shown in Fig. 5d, It is noticed that the instantaneous values of *DF*_*b*_ = *DF*_*c*_ =  + 1.0 before and during the fault time, while *DF*_*a*_ =  + 1.0 before the fault inception and *DF*_*a*_ = − 1.0 at discrete intervals during the external fault time due to the CT saturation event. Moreover, *DF*_*a*_ is changed from the value of 1.0 to − 1.0 and vice versa during the fault time, but the majority of fault time *DF*_*a*_ = 1.0. Thus, *DF*_*a*_ indicates the occurrence of CT saturation state for the ‘*A*’ phase current signal, while the values of *DF*_*b*_ and *DF*_*c*_ affirm that there is no CT saturation condition for the two current signals of ‘*B*’ and ‘*C*’ phases.

Figure 6a shows the average values of the three-phase directionality factors (*DF*_*av*_*, DF*_*bv*_, and *DF*_*cv*_) for each data window, whose interval is selected as a half-cycle. As seen in Fig. 6a, in the case of external fault with CT saturation extent, the average values (*DF*_*av*_*, DF*_*bv*_, and *DF*_*cv*_) of the three directionality factors are positive values; and the average values of *DF*_*a*_ = *0.88* (during the first window after the fault time inception). These values prove that the fault type is external with ‘*A*’ phase CT saturation. This is because the three directionality factors (*DF*_*av*_*, DF*_*bv*_, and *DF*_*cv*_) are positive, and the values of *DF*_*a*_ exceed 1.0. Additionally, the calculated differential currents (*i*_*da*_*(k)*, *i*_*db*_*(k)*, and *i*_*dc*_*(k)*) are below the set characteristic (i.e., the operating points are located within the blocking area) for the three phases of the protected element during the first window of the fault time inception. Figure 6b depicts the calculated maximum form and ripple factors, *FF*_*i*_ and *RF*_*i*_ estimated for the ‘*A*’ phase of the *TL*_*2*_ circuit. As shown in Fig. 6b, *FF*_*i *_*≈ *1.57 and *RF*_*i *_*≈ *1.21 during the pre-fault and the steady-state fault period (i.e., fault current without DC component extent). The two factors *FF*_*i*_ and *RF*_*i*_ become greater than 1.65 and 1.31, respectively, during the distorted portions and the large DC component extent in the saturated secondary current; and their values are the same pre-fault values during the unsaturated portions for the ‘*A*’ phase secondary current of the faulted circuit. Figure 6c plots the estimated form and ripple factors, *FF*_*ix*_ and *RF*_*ix*_, (estimated for ‘*A*’ phase of the TL_2_ circuit). As shown in Fig. 6c, *FF*_*ix *_*≈ *1.30 and *RF*_*ix *_*≈ *0.83 during the pre-fault and the steady-state fault conditions. The two factors (*FF*_*ix*_ and *RF*_*ix*_) are less than 1.26 and 0.76, respectively, during the saturated portions and the large DC component. Their values resemble those of the pre-fault values during the free saturation portions of the ‘*A*’ phase current signal. Figure 6d displays the estimated restraint factors (*K*_*s1*_ and *K*_*s2*_) of the adaptive differential relay, where their numerical values are modified with the changes of CT saturation level and the DC component content of the fault current. As mentioned before, the two slopes (*K*_*s1*_ and *K*_*s2*_) are based on the calculated form and ripple factors (*FF*_*ix*_ and *RF*_*ix*_), respectively.

Figures 7a–c illustrate the instantaneous differential and restraining current signals (*i*_*ds*_ and *I*_*d0*_ + *K*_*s2*_ × *i*_*rs*_) for the *A, B* and *C* phases of the protected busbar (*BB*_*1*_), respectively. Figure 7a shows the values of *i*_*da *_*≈ *0 (which correspond to *RF*_*i *_*≈ *1.21) before the fault time inception and during the free saturation portions of the fault current, while the values of *i*_*da*_ become larger (which correspond to the *RF*_*i*_ values ≥ 1.31) during the saturated portions for the ‘*A*’ phase fault current signal. Figures 8a–c present the average values of the differential and restraining current signals (*i*_*ds*_ and *I*_*d0*_ + *K*_*s2*_ × *i*_*rs*_) of the *BB*_*1*_ three phases, which are estimated for each half-cycle data window of the current signal. The mathematical formula of the adaptive characteristic (*I*_*d0*_ + *K*_*s2*_ × *i*_*rs*_) includes three variables as follows: the pickup value of differential current (*I*_*d0*_), the adaptive slope (*K*_*s2*_*)*, and the restraining current signal* (i*_*rs*_). In this case study, it is observed that the three-phase differential currents (*i*_*da*_, *i*_*db*_ and *i*_*dc*_) are lower than the three-phase restraining currents ((*I*_*d0*_ + *K*_*s2*_ × *i*_*ra*_), (*I*_*d0*_ + *K*_*s2*_ × *i*_*rb*_), and (*I*_*d0*_ + *K*_*s2*_ × *i*_*rc*_)), respectively, as depicted in Figs. 8a–c. Thus, this makes the operating points concentrated inside the stabilizing zone of the relay operating characteristic. As a result, the proposed relay prevents isolating the CBs of the busbar (*BB*_*1*_). Figure 8d plots the three-phase directionality factors (*DF*_*a*_*, DF*_*b*_, and *DF*_*c*_) in the instance of the external fault without CT saturation and *R*_*b*_ = 0.5 Ω for each CT of the protected busbar (*BB*_*1*_). It is clear that *DF*_*a*_ = *DF*_*b*_ = *DF*_*c*_ = 1.0 during the time periods of the normal operation and the external fault without CT saturation condition (as shown in Fig. 8d). Therefore, the protection scheme holds a trip signal to low (0) in the case of external fault without/with CT saturation presence.

From the extensive simulation results, it is obvious that the adaptive differential current relay, based on the estimated form and ripple factors of the measured current signals is able to determine the instant fault onset, detect CT saturation conditions, identify the fault location zone (whether it is an internal or external fault), and efficiently adjust its tripping characteristics according to the change in the CT saturation level and the DC component. Moreover, it has the ability to differentiate between external faults with and without CT saturation extent, and it can define the CT saturation condition, identify the distorted and undistorted portions of the current waveform, assess the CT saturation level, and select which feeder CT is saturated.

(**c**) The ‘*C*’ phase secondary currents of the four feeders. (**d**) The ‘*A*’ phase primary and secondary (referred to primary side) currents of the third feeder (*TL*_*2*_ = the faulted feeder). (**a–d**) Simulation current signals for case study 1.

### *Case study 2: internal SLG fault with CT saturation*

**Fig. 9:**
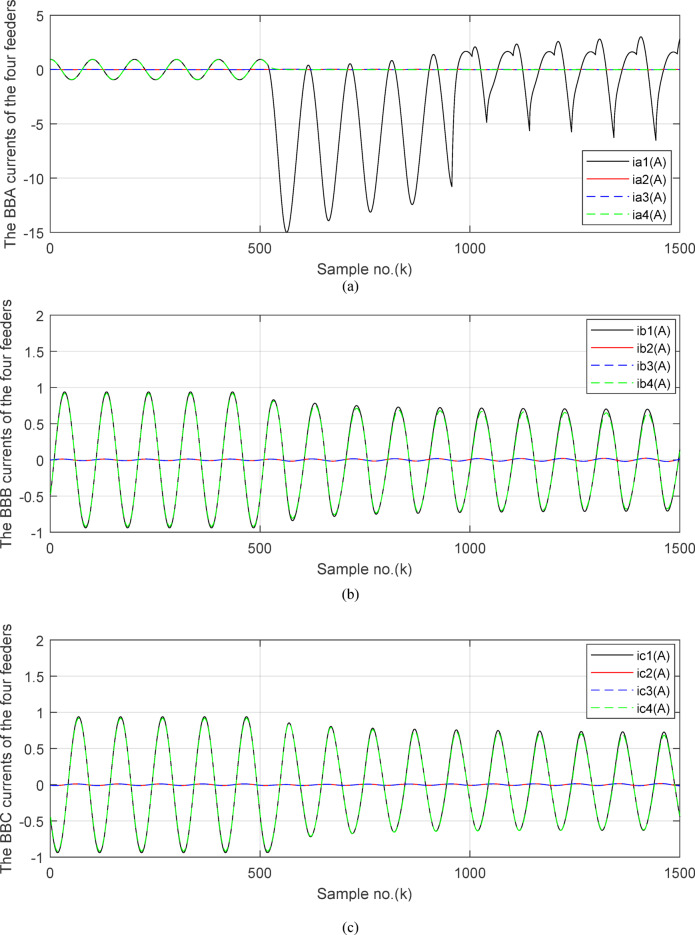

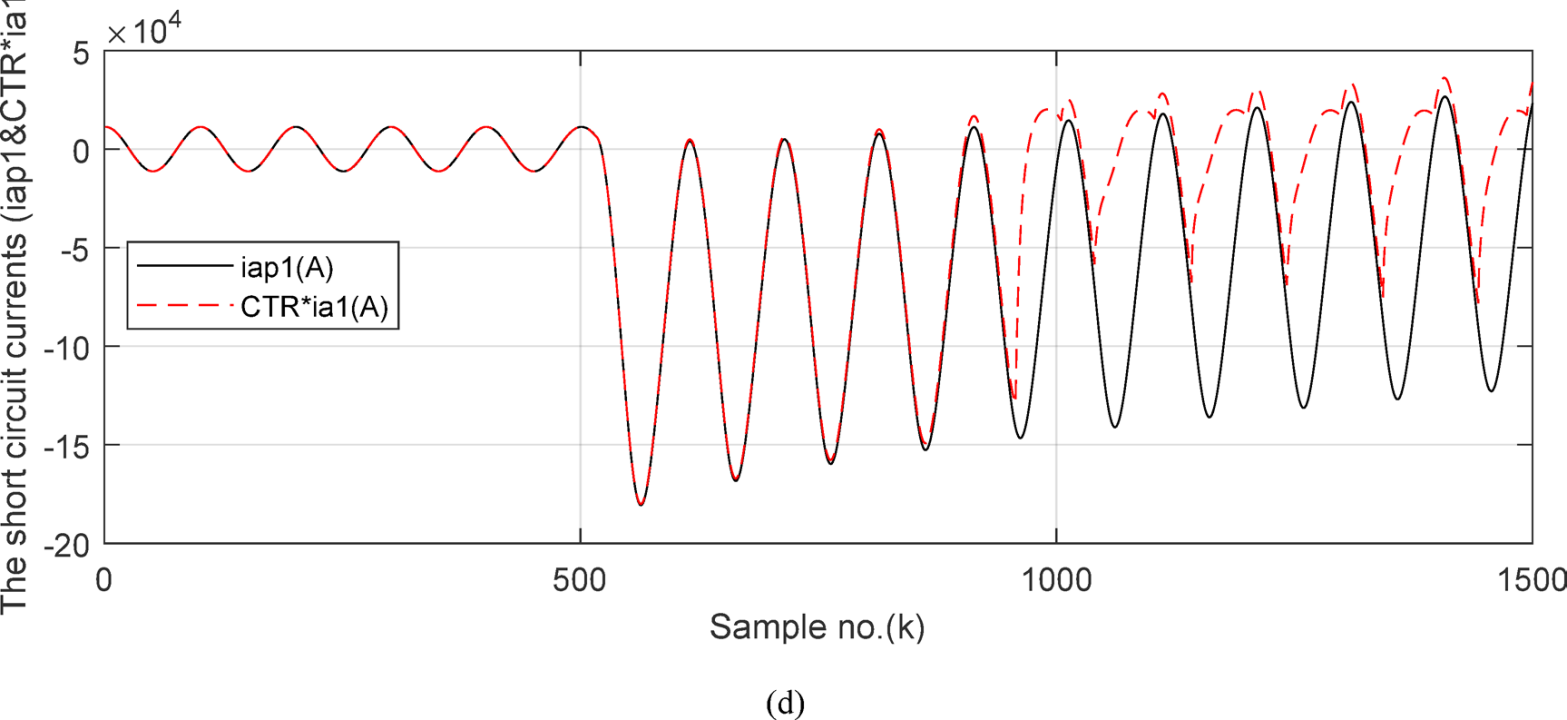
(**a**) The ‘*A*’ phase secondary currents of the four feeders. (**b**) The ‘*B*’ phase secondary currents of the four feeders. (**c**) The ‘*C*’ phase secondary currents of the four feeders. (**d**) The ‘*A*’ phase primary and secondary (referred to primary side) currents of the first branch (the generator circuit). (**a**–**d**) Simulation current signals for case study 2.

**Fig. 10:**
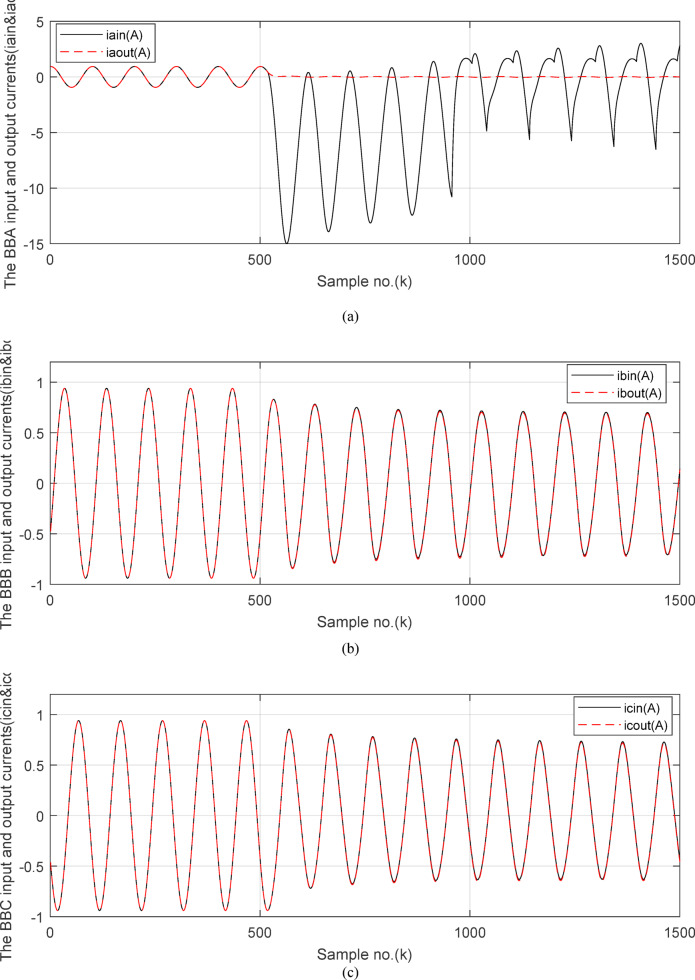

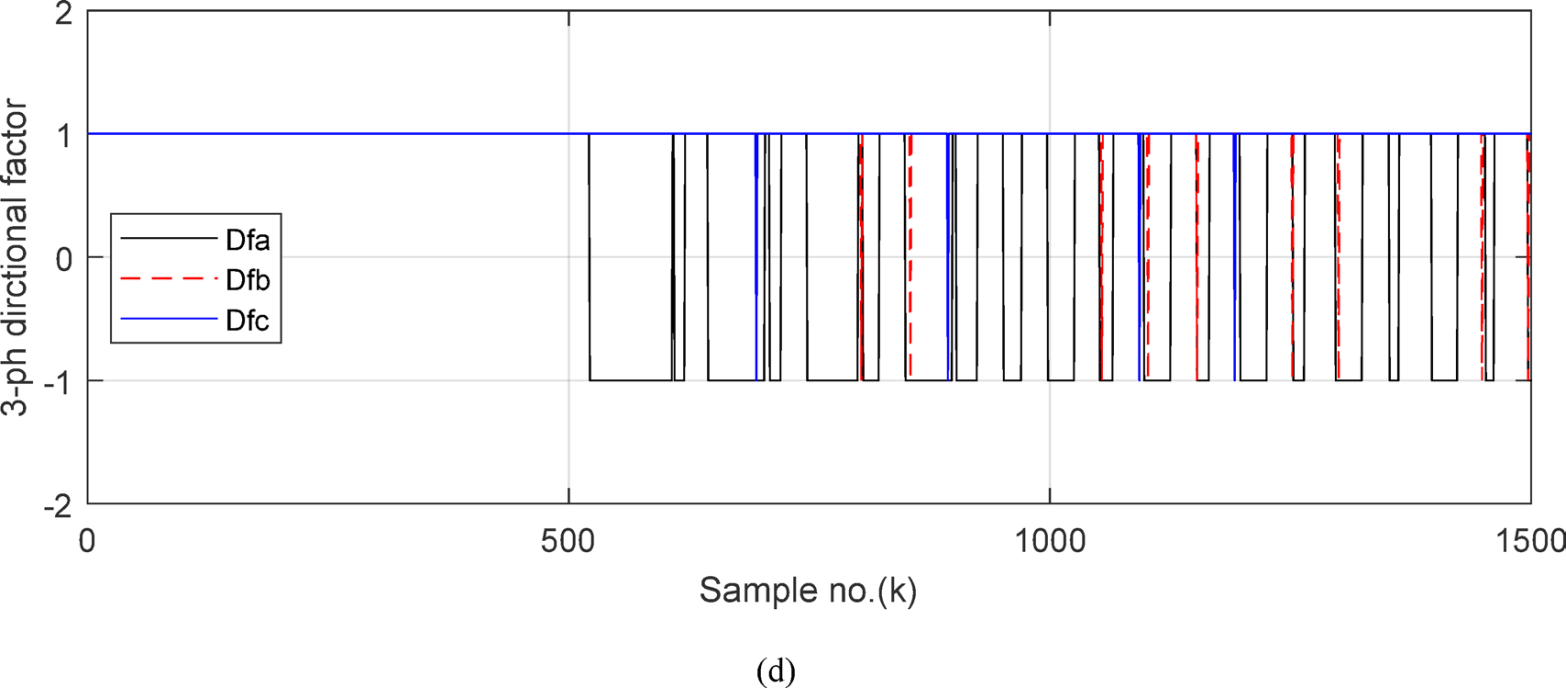
(**a**) The input and output secondary current signals (*i*_*ain*_ and *i*_*aout*_) for the ‘*A*’ phase of *BB*_*1*_. (**b**) The input and output secondary current signals (*i*_*bin*_ and *i*_*bout*_) for the ‘*B*’ phase of *BB*_*1*_. (**c**) The input and output secondary current signals (*i*_*cin*_ and *i*_*cout*_) for the ‘*C*’ phase of *BB*_*1*_. (**d**) The instantaneous values of three phases directionality factors (*DF*_*a*_*, DF*_*b*_ and *DF*_*c*_) in the case of internal fault. (**a**–**d**) simulation current signals and the directionality factors for case study 2.

**Fig. 11:**
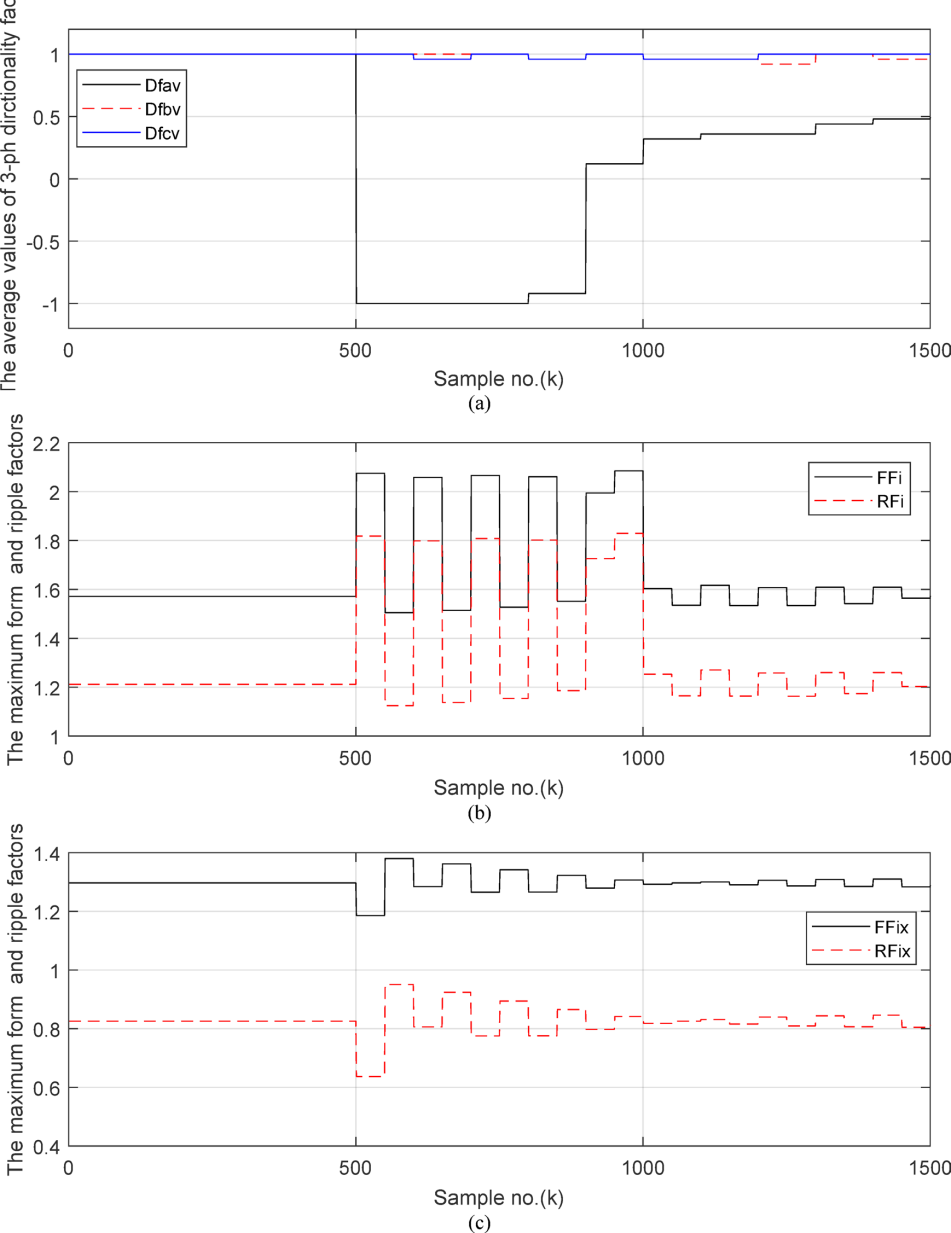

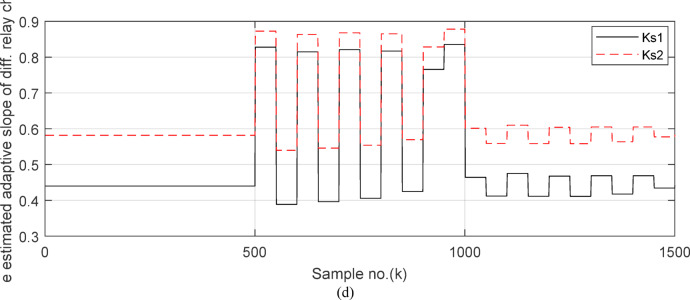
(**a**) The average values of three phases directionality factors (*DF*_*av*_*, DF*_*bv*_ and *DF*_*cv*_) in the case of internal *SLG* fault with CT saturation. (**b**) The calculated maximum form and ripple factors (*FF*_*i*_ and *RF*_*i*_). (**c**) The calculated form and ripple factors (*FF*_*ix*_ and *RF*_*ix*_). (**d**) The calculated operating characteristic slope of differential relay (*K*_*s1*_ and *K*_*s2*_). (**a**–**d**) Response of adaptive busbar differential relay for case study 2.

**Fig. 12:**
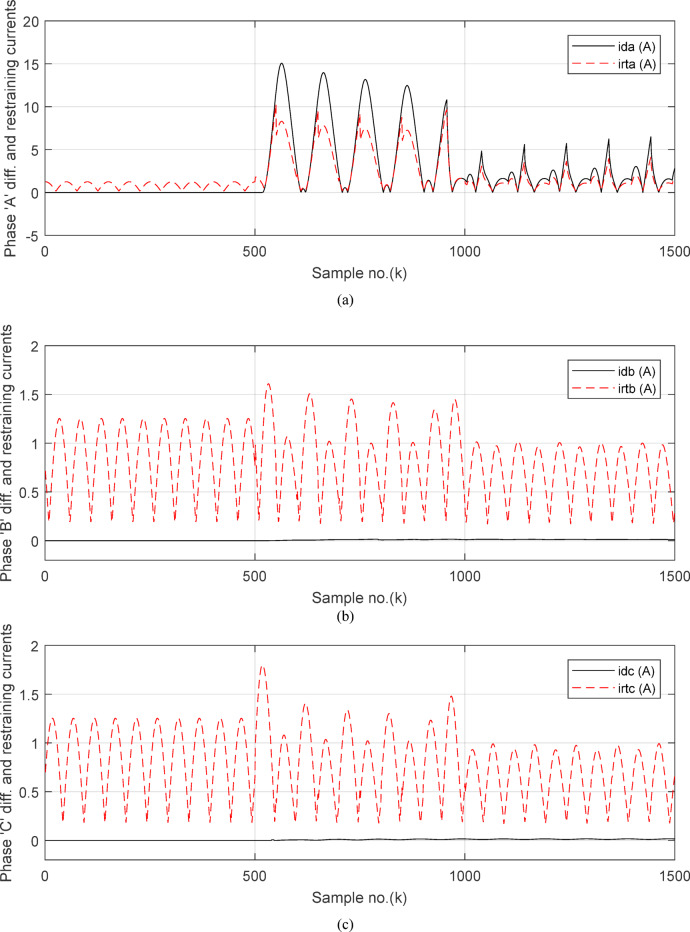
(**a**) The instantaneous differential and restraining current signals for ‘*A*’ phase of *BB*_*1*_ (*i*_*da*_ and *I*_*d0*_ + *K*_*s2*_ × *i*_*ra*_). (**b**) The instantaneous differential and restraining current signals for ‘*B*’ phase of *BB*_*1*_ (*i*_*db*_ and *I*_*d0*_ + *K*_*s2*_ × *i*_*rb*_). (**c**) The instantaneous differential and restraining current signals for ‘*C*’ phase of *BB*_*1*_ (*i*_*dc*_ and *I*_*d0*_ + *K*_*s2*_ × *i*_*rc*_). (**a**–**c**) The calculated instantaneous differential and restraining current signals for case study 2.

**Fig. 13:**
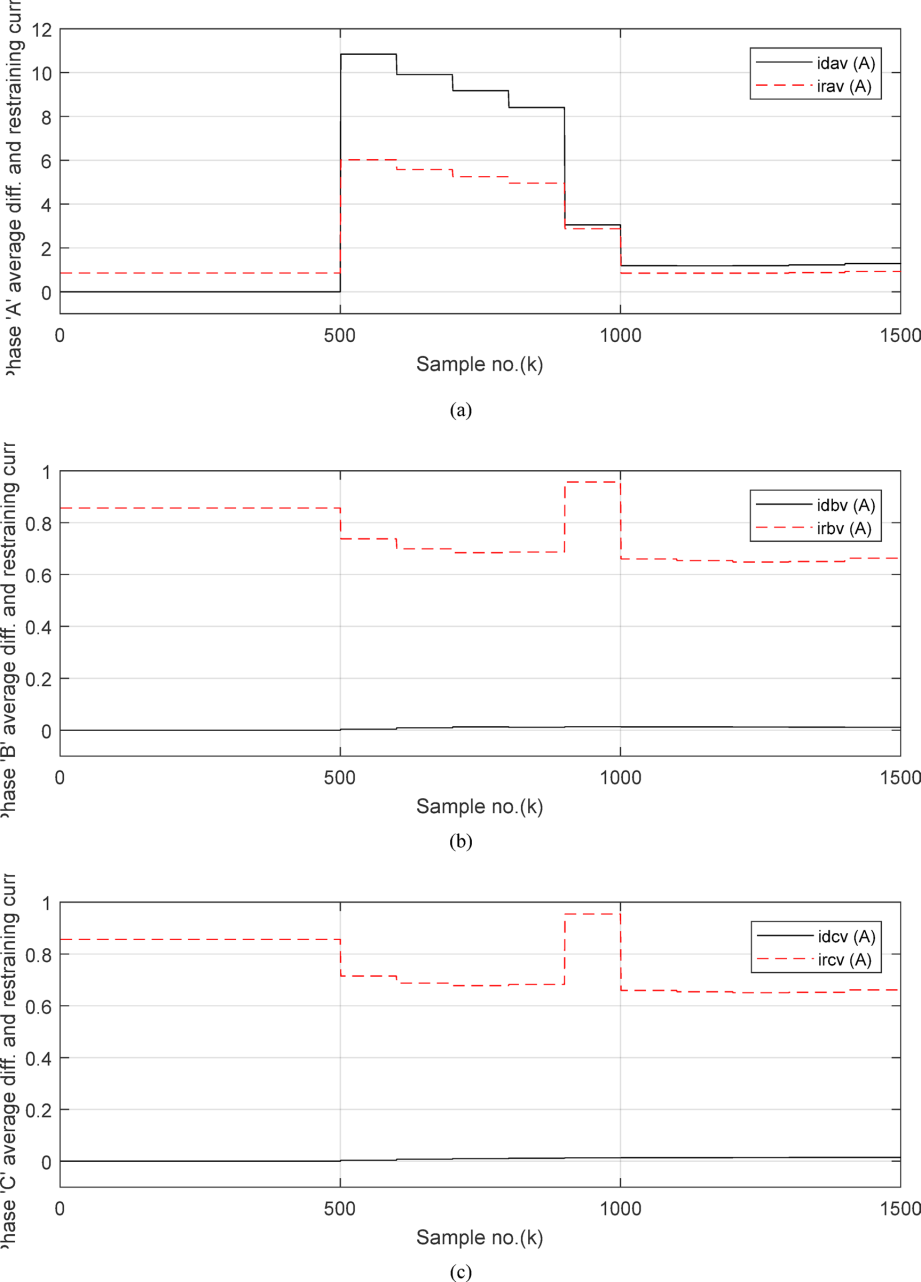
(**a**) The average value of differential and restraining current signals (*i*_*da*_ and *I*_*d0*_ + *K*_*s2*_ × *i*_*ra*_) for the ‘*A*’ phase of *BB*_*1*_. (**b**) The average value of differential and restraining current signals (*i*_*db*_ and *I*_*d0*_ + *K*_*s2*_ × *i*_*rb*_) for the ‘*B*’ phase of *BB*_*1*_. (**c**) The average value of differential and restraining current signals (*i*_*dc*_ and *I*_*d0*_ + *K*_*s2*_ × *i*_*rc*_) for the ‘*C*’ phase of *BB*_*1.*_ (**a**–**c**) The calculated average differential and restraining current signals for case study 2.

This case examines the performance of the proposed scheme in the event of internal *SLG (A*-*G*) fault with CT saturation condition. The fault is without fault resistance (*R*_*f*_ = 0.0 *Ω*), and it is placed at the point *F*_*2*_ on *BB*_*1*_. In this case study, the operating conditions of the power system parameters are the same as those of in case 1, except that the *CTR* of the CT for each feeder is changed from 1000/1 to 12,000/1, and the CT burden is 0.5 + j0.0 Ω for each CT. Figures 9, 10, 11, 12 and 13 present the simulation results for case study 2. Figure 9a–c depict the three-phase secondary currents for the four branches connected to *BB*_*1*_ in the event of the internal *SLG (A*-*G*) fault with CT saturation. Figure 9d shows the primary and saturated secondary (referred to as primary side) currents for the ‘*A*’ phase of the generator circuit. It is noticed that the current quantity (for the ‘*A*’ phase of the generator circuit) during the fault interval is higher than the pre-fault current quantity. Its magnitude is greater than 7 × *I*_*n*_, and the current signal is accompanied by a large DC component. For the ‘*A*’ phase fault current of the generator circuit, the large magnitude of the fault current with the high DC component cause the distorted secondary current, as depicted in Fig. 9d. In this case, the CT saturation situation appears after five cycles from the fault time inception. As a consequence, the protection scheme should isolate rapidly the faulted *BB*_*1*_ to avoid the CT saturation. In this instance, the internal fault associated with the presence of CT saturation is analyzed and discussed.

Figures 10a–c offer the estimated input and output current signals of the *A, B* and *C* phases for the protected busbar (*BB*_*1*_), respectively. The input and output current signals of the ‘*B*’ and ‘*C*’ phases are in phase and equal before and during the fault time, while the input and output current signals for the ‘*A*’ phase of the busbar (*BB*_*1*_) are identical before the fault span and their values are unsymmetrical during the fault interval. Figure 10d demonstrates the instantaneous values of the three-phase directionality factors (*DF*_*a*_*, DF*_*b*_, and *DF*_*c*_) in the incidence of the internal fault accompanied by CT saturation. As obvious in Fig. 10d, the *DF*_*a*_ = *DF*_*b*_ = *DF*_*c*_ = 1.0 during the normal operation period, while the *DF*_*a*_ = *− 1.0* and *DF*_*b*_ = *DF*_*c*_ =  + *1.0* during the first data window after the commencement of the fault time. Figure 11a reveals the average values of the three-phase directionality factors (*DF*_*av*_*, DF*_*bv*_, and *DF*_*cv*_) computed for each data window of the fault current. The three-phase directionality factors assure that the fault location is within the *BB*_*1*_ protection zone, and its type is *SLG (A-G)* fault. This is because the values of the factor (*DF*_*av*_) are negative, while the values of the factors (*DF*_*bv*_ and *DF*_*cv*_) are positive. Moreover, the estimated differential current (*i*_*da*_*(k)*) for the ‘*A*’ phase of the protected busbar is above the set characteristic (i.e., the operating points are located in the tripping area within the differential relay characteristic). Figure 11b depicts the calculated maximum form and ripple factors (*FF*_*i*_ and *RF*_*i*_ ) for the ‘*A*’ phase of the generator circuit. As shown in Fig. 11b, *FF*_*i *_*≈ *1.57 and *RF*_*i *_*≈ *1.21 during the pre-fault condition (i.e., during the normal operation period). The two factors (*FF*_*i*_ and *RF*_*i*_) become greater than 1.65 and 1.31, respectively, during the interval of asymmetrical fault current (i.e., the period of DC component extent). Figure 11c illustrates the estimated form and ripple factors (*FF*_*ix*_ and *RF*_*ix*_) calculated for the ‘*A*’ phase current of the generator circuit. As illustrated in Fig. 11c, *FF*_*ix *_*≈ *1.30 and *RF*_*ix *_*≈ *0.83 during the normal operation period, while the two factors become less than 1.26 and 0.76, respectively, during the large DC component. Figure 11d displays the suitable operating characteristic slopes (*K*_*s1*_ and *K*_*s2*_) of the adaptive differential current relay for case study 2.

Figures 12a-c illustrate the instantaneous differential and restraining current signals (*i*_*ds*_ and *I*_*d0*_ + *K*_*s2*_ × *i*_*rs*_) for the three phases of the protected busbar (*BB*_*1*_) in the event of the internal *SLG* (*A-G*) fault with CT saturation. Figure 12a shows the values of *i*_*da *_*≈ *0.0 (which correspond to the values of *RF*_*i *_*≈ *1.21) before the fault time inception, while the values of *i*_*da*_ become larger (which correspond to the *RF*_*ix*_ values that are greater than 1.31) during the fault time. The values of *i*_*db *_*≈ i*_*dc *_*≈ *0.0 before and during the fault time, as depicted Fig. 12b–c. Figures 13a–c present the average values of the differential and restraining current signals (*i*_*ds*_ and *I*_*d0*_ + *K*_*s2*_ × *i*_*rs*_) for the three phases of the protected busbar (*BB*_*1*_). In this case study, it is clear that the three-phase differential currents (*i*_*da*_, *i*_*db*_ and *i*_*dc*_) are lower than the three-phase restraining currents ((*I*_*d0*_ + *K*_*s2*_ × *i*_*ra*_), (*I*_*d0*_ + *K*_*s2*_ × *i*_*rb*_), and (*I*_*d0*_ + *K*_*s2*_ × *i*_*rc*_)), respectively, as depicted in Fig. 13a–c. Consequently, the operating points are located inside the tripping zone of the relay characteristic curve. Hence, this assures that the fault location is internal, and the fault type is *SLG* (*A-G*). Thus, a tripping signal is sent to the CBs for isolating the faulted busbar (*BB*_*1*_) from the remaining power system. This is accomplished by disconnecting the CBs of the four branches connected to the busbar (*BB*_*1*_). The protection scheme sets the tripping signal to high (1) in the situation of the internal fault.

To further investigate the numerical accuracy and robustness of the proposed method, many study cases have been simulated under the influence of normal operation with varying electrical loads and running power factor. The load changes from 1.0 to 20% of the rated load (with step 1.0%) at running power factor = 0.85, and the running power factor is altered from 0.8 to 1.0 (with step 0.1) at the same rated real power. It is also examined under the effect of different fault conditions, including fault zones (internal and external), faulty phases (A-G, B-G, C-G, A-B-G, B-C-G, C-A-G, A-B, B-C, C-A, and A-B-C-G), fault resistances (*R*_*f*_), fault inception times (*t*_*f*_), fault locations on TL_2_ length, and various CT saturation levels. The fault resistance (*R*_*f*_) changes from 0.2 to 200 Ω (with step 0.2 Ω), and the fault inception time (*t*_*f*_) changes from 0.100 to 0.120 Sec (with step 0.001 Sec) with varying the faulty phase(s). The level of the CT saturation could be elevated by reducing the Current Transformer Ratio (CTR) and/or increasing the CT burden (*R*_*b*_).

The performance of the Adaptive Differential Overcurrent Relay (ADOCR) for the diverse scenarios is given in Table 3. The table reveals that the developed ADOCR responds to the instance of internal faults, while it remains inactive in the situation of normal operations or external faults with/without CT saturation.

**Table 3:** The performance of the proposed ADOCR under the influence of different operating and fault conditions.

Fault type	Fault location	fault inception time, *t*_*f*_(in Sec)	Fault resistance, *R*_*f*_(in Ω)	Current transformer ratio (CTR)	CT burden, *R*_*b*_(in Ω)	The proposed ADOCR response (Tripping/Blocking)
	F_2_ is the location of internal fault (at BB_1_).F_1_ is the location of external fault (at 25% of TL_2_ length, starting from the point of BB_1_)					
Internal SLG (A-G) fault without CT saturation	F_2_	0.103	0.0	12,000/1	0.5 + j0.0	Tripping
Internal SLG (A-G) fault with CT saturation						Tripping
Internal DLG (A-B-G) fault without CT saturation						Tripping
Internal DLG (A-B-G) fault with CT saturation						Tripping
Internal DL (A-B) fault without CT saturation						Tripping
Internal DL (A-B) fault with CT saturation						Tripping
Internal 3LG (A-B-C-G) fault without CT saturation						Tripping
Internal 3LG (A-B-C-G) fault with CT saturation						Tripping
External SLG (A-G) fault without CT saturation	F_1_					Blocking
External DLG (A-B-G) fault without CT saturation						Blocking
External DL (A-B) fault without CT saturation						Blocking
External 3LG (A-B-C-G) fault without CT saturation						Blocking
External SLG (A-G) fault with CT saturation				1000/1	The CT burden for all feeders is *R*_*b*_ = 0.5 + j0 Ω except the CT burden *R*_*b*_ = 500 Ω for ‘A’ phase CT of the third branch (TL_2_)	Blocking
External DLG (A-B-G) fault with CT saturation					The CT burden for all feeders is *R*_*b*_ = 0.5 + j0 Ω except the CT burden *R*_*b*_ = 500 Ω for ‘A and B’ CTs of the third branch (TL_2_)	Blocking
External DL (A-B) fault with CT saturation						Blocking
External 3LG (A-B-C-G) fault with CT saturation					The CT burden for all feeders is *R*_*b*_ = 0.5 + j0 Ω except the CT burden *R*_*b*_ = 500 Ω for ‘A, B and C’ CTs of the third branch (TL_2_)	Blocking
Internal SLG (A-G) fault without CT saturation	F_2_	*t*_*f*_ changes from 0.100 to 0.120 Sec (with step 0.001 Sec)	*R*_*f*_ changes from 0.2 to 200 Ω (with step 0.2 Ω)	12,000/1	0.5 + j0.0	Tripping
Internal DLG (A-B-G) fault without CT saturation						Tripping
Internal DL (A-B) fault without CT saturation						Tripping
Internal 3LG (A-B-C-G) fault without CT saturation						Tripping
External SLG (A-G) fault without CT saturation	F_1_					Blocking
External DLG (A-B-G) fault without CT saturation						Blocking
External DL (A-B) fault without CT saturation						Blocking
External 3LG (A-B-C-G) fault without CT saturation						Blocking
Normal operation at the rated load and running power factor = 0.85	Not applicable	Not applicable	Not applicable	12,000/1	0.5 + j0.0	Blocking
Normal operation with load change from 1.0% to 20% of the rated load (with step 1.0%) at running power factor = 0.85	Not applicable	Not applicable	Not applicable	12,000/1	0.5 + j0.0	Blocking
Normal operation with the change of the running power factor from 0.8 to 1.0 (with step 0.1) at the same rated real power	Not applicable	Not applicable	Not applicable	12,000/1	0.5 + j0.0	Blocking

Table 4 presents the assessment of the protection characteristics for the proposed scheme. The ratios of the protection algorithm’s dependability, security, reliability, and accuracy exceed 99.40%, as indicated by Table 4. Finally, a comparative evaluation of the proposed ADOCR with the existing ADOCRs is held, which clearly indicates the superiority of the proposed ADOCR, as shown in Table 5.

**Table 4:** Assessment of the protection characteristics.

The busbar state	Number of scenarios	Malfunction times
The total number of internal faults	1401	5.0 (The algorithm failed to trip in 5.0 instances of the internal faults as a result of the very high fault resistances.)
The total number of external faults	1023	4.0 (The algorithm sent an incorrect trip in 4.0 instances of the external faults due to the largest CT saturation degree, which results from the high fault current, CT burden, and DC component combined in these cases.)
The total number of normal operations	41.0	0.0
The total number of trips	1401—5 + 4 = 1400	4.0
Protection characteristics evaluation	Qn_1_ = The total number of scenarios = 1401 + 1023 + 41	= 2465
	Qn_2_ = The total number of trips = 1401—5 + 4	= 1400
	Qn_3_ = The number of correct trips = 1401—5	= 1396
	Qn_4_ = The number of tripping failures (in some cases of internal faults)	= 5.0
	Qn_5_ = The number of desirable trips (due to all cases of internal faults) = Qn_3_ + Qn_4_	= 1396 + 5 = 1401
	Qn_6_ = The number of incorrect trips (due to some cases of external faults) = Qn_2_–Qn_3_	= 1400–1396 = 4.0
	Qn_7_ = Qn_5_ + Qn_6_ = Qn_2_ + Qn_4_	= 1401 + 4 = 1400 + 5 = 1405
	$\% DR = \frac{{{\text{Qn}}3 }}{{{\text{Qn}}5}} \times 100$	$= \frac{1396}{{1401}} \times 100 = 99.64\%$
	$\% SR = \frac{{{\text{Qn}}3}}{{{\text{Qn}}2}} \times 100$	$= \frac{1396}{{1400}} \times 100 = 99.71\%$
	$\% RR = \frac{{{\text{Qn}}3}}{{ {\text{Qn}}7}} \times 100$	$= \frac{1396}{{1405}} \times 100 = 99.40\%$
	$\% AR = \frac{{{\text{Qn}}1 - {\text{Qn}}4 - {\text{Qn}}6}}{{{\text{Qn}}1}} \times 100$	$= \frac{2465 - 5 - 4 }{{2465}} \times 100 = 99.63\%$

DR = Dependability ratio, SR = Security ratio, RR = Reliability ratio, and AR = Accuracy ratio.

**Table 5:** Comparison between alienation/correlation coefficients-based ADOCRs and form/ripple factors-based ADOCR.

Item	Published ADOCRs based on the alienation/correlation coefficients^12–14^	Proposed ADOCR based on the form/ripple factors
1. Main concept	Adaptive differential relaying scheme based on the alienation^12,13^ and correlation^14^ coefficients estimated between the input and output current signals of the protected power system element	Adaptive differential relaying scheme based on the form and ripple factors computed for the measured current signals
2. Operating characteristic type	It is described as a variable-bias digital differential relay for protecting a power system element. The amount of bias is varied linearly with the values of alienation^12,13^ and correlation^14^ coefficients calculated between the calculated input and output current signals of the power grid element The operating characteristic includes three zones as follows: blocking zone, adaptive zone, and tripping zone	It is described as a variable-bias digital differential relay for protecting power system elements. The amount of bias is varied linearly with the values of form and ripple factors calculated for the measured currents The operating characteristic includes three zones as follows: blocking zone, adaptive zone, and tripping zone
3. Restraint factor (Characteristic slope)	It is based on a linear equation derived from the alienation^12,13^ and correlation^14^ coefficients calculated between the summation of input currents and the summation of output currents for the protected equipment	It is based on a linear mathematical formula derived from the form and ripple factors computed for the measured phase current signals
4. Feeder selection of saturated CT	It has the ability to determine CT saturation condition and its assessment, but it fails to find which feeder CT is saturated	It is able to identify CT saturation condition, assess its level, and select which feeder CT is saturated
5. Multi-functions	The alienation^12,13^ and correlation^14^ algorithms integrated with the differential current scheme can be used to carry out various protection functions, such as Current fault detector, Faulty phase selection, Fault classifier, Fault direction discrimination, CT saturation detector, CT saturation assessment, and Adaptive tripping characteristics during the time periods of CT saturation	The differential current scheme can be used to carry out various protection functions, including Current fault detector, Faulty phase selection, Fault classifier, and Fault direction discrimination, Whereas the form and ripple factors can be used for CT saturation and DC component detector, CT saturation and DC component assessment, and Adaptive tripping characteristics during the time periods of CT saturation and DC component current
6. Data window size	The data window size can be modified and selected to be 1/8. 1/4, 1/2, 3/4, or one cycle	
7. Relay stability	The relay is restrained from operation on CT saturation in the case of the external faults. The proposed relay is stable on those heavy through-faults on which a fixed-bias relay may operate The stability is characterized by the efficiency of the relay’s method of identifying the electrical system’s operating conditions	The relay is restrained from operation on CT saturation and DC component in the case of external faults. The proposed relay is stable on those heavy through-faults on which a fixed-bias relay may operate
8. Relay reliability	It is characterized as high reliability because it is immune to fault time duration, fault inception angle, fault location, or fault type issues. Besides, it is able to detect all types of shunt faults The suggested protective relay satisfies the requirements of protection reliability (i.e., protection dependability and security) for operation. The reliability is determined primarily by the absence of failures in the operation of the protective relays. However, a compromise in the relay settings is still required to make coordination between protection stability and dependability, as well as between protection security and sensitivity	It is characterized as high reliability because it is immune to fault time duration, fault inception angle, fault location, or fault type issues. Besides, it is able to detect all types of shunt faults The suggested protective relay satisfies the requirements of reliability (i.e., dependability and security) for operation
9. Data synchronization system	The proposed and existing protection schemes require no data synchronization system (it needs only fiber optics)	
10. Digital low-pass filter	The selected data window size, used to calculate the alienation^12,13^ and correlation^14^ coefficients and differential and restraining currents, performs the functional role of the digital low-pass filter	The selected data window size, used to compute the form and ripple factors, and differential and restraining currents, behaves as the task of the digital low-pass filter
11. Fields of relay applications	The proposed method can be advantageous with wide application scope, where it is useful in Smart Grids (SGs) and Substation Automation Systems (SAS). Moreover, it can be used for various equipment (including synchronous generators, power transformers, AC motors, underground cables, and short transmission lines) with different voltage levels	The proposed method can be advantageous with wide application scope, where it is useful in Smart Grids (SGs) and Substation Automation Systems (SAS). Moreover, it can be used for various equipment (including synchronous generators, power transformers, AC motors, underground cables, and short transmission lines) with different voltage levels

The obtained results demonstrate the following information:

1. The causes of the presence of CT saturation severity are as follows:
  - Increase of CT secondary burden (*R*_*b*_),
  - Increase of CT primary fault current (due to a reduction in the fault resistance, *R*_*f*_),
  - Increase of asymmetry in the primary fault current (due to the presence of DC components), and
  - Low current transformer ratio (CTR).
2. The proposed relay of the adaptive percentage differential type has some immunity to its malfunction in the case of severe external faults (with/without CT saturation extent and DC component content) because its operating characteristic requires a substantial ratio of operating current to restraining current.
3. In conventional differential relays, the presence of harmonics (as a result of DC components, CT saturation, or both together) may delay or prevent operation in the event of the severe internal faults. It is customary to include a high-set, unrestrained overcurrent unit in these relays. The present method addresses this issue.

## Protection scheme assessment

### Protection characteristics evaluation

In this study, the scenarios of internal and external faults were 1401 and 1023, respectively. There were 1400 trips in total, of which 4.0 were incorrect. Out of all scenarios, the algorithm failed to trip in 5.0 instances. The algorithm was restrained 41.0 times without any malfunctions when the system was operating normally. Table 4 illustrates the estimated dependability (DR), security (SR), reliability (RR), and accuracy (AR) of the algorithm under the influence of disparity of internal and external faults (encompassing three line, double line, double line-to-ground, and single line-to-ground faults with/without arc resistances, as well as the error of current transformers)^39–45^. The table demonstrates that the estimated values of DR, SR, RR, and AR are greater than 99.40%.

### Protection scheme features

The main achievements of the adaptive differential protection are enumerated as follows:

1. It is suitable for application in digital relays,
2. It can be implemented simply because it utilizes the simplest technique of signal processing compared to the other existing schemes,
3. It is secure to the external faults accompanied by light/heavy CT saturation,
4. It has an adequate stable response for varying operating and fault conditions,
5. It has a fast response, as the operating time taken by this method is a sub-cycle,
6. It is effective, accurate and reliable,
7. It can operate online for all voltage levels of traditional and smart grids,
8. It can be used for protecting different power system elements (such as synchronous generators, power transformers, busbars, and short transmission lines),
9. It is able to apply various sizes of moving data windows for signal processing; thus, the relay operation speed can be controlled,
10. It can use various slopes criteria to execute the concept of adaptive differential relay characteristics,
11. It is useful to detect the saturated current signals, assess the severity level of CT saturation, evaluate the content of DC components in the fault current signals, and adapt the differential relay characteristics,
12. No need for selecting dual characteristic slopes or a fixed slope where the modified relay is provided with the adaptive characteristic slope,
13. Three-phase current measurements (for the two terminals of the protected equipment) are sufficient for processing the proposed algorithm; the specifications of power system elements and current transformers are not required,
14. The technique can be implemented practically,
15. The improved differential overcurrent technique develops various protection functions, such as current fault detector, faulty phase selection, fault classifier, fault direction discrimination, CT saturation detector, CT saturation assessment, and differential relay with adaptive tripping characteristics based on the form/ripple factors for the measured current signals to avoid the impacts of the current transformer saturation and the DC components.

### Critical comparisons

Table 5 presents a comparison between the existing adaptive differential current relays (ADOCRs) based on alienation^12,13^ and correlation^14^ coefficients for current signals, and the proposed adaptive differential current relay (ADOCR) based on form/ripple factors for the current signals measured at the two terminals of the protected busbrs.

## Conclusions

This paper has presented a protection algorithm for adaptive differential relay characteristics, taking into account the levels of CT saturation and DC component content of the current signals measured at the two ends of the protected equipment. The restraining factor has been adjusted online using the values of form and ripple factors calculated for the measured currents. The form and ripple factors, in addition to the three-phase directionality factors, have been used for CT saturation detection. These factors are useful for regulating a restraint signal in order to prevent the relay operation during external faults with/without CT saturation conditions and accelerate the relay operation in the case of large internal fault currents with high DC components. The developed adaptive protection includes changing the differential relay curve online using the form and ripple factors estimated for the equipment currents. As a result, it improves protection stability and security in the event of close external faults with/without CT saturation and increases protection sensitivity and dependability in the event of internal faults with high DC components. On a typical three-phase power system, the proposed technique has been examined under diverse operating and abnormal states (including fault types, fault locations, fault resistances, fault inception angles, CT ratios and burdens, and CT saturation levels). The ATP simulator has been used to simulate the power system under study with real data of its parameters, while MATLAB software has been used to process the adaptive differential protection algorithm. The simulation results have demonstrated noticeably better relay performance when compared to traditional relays that lack adaptive features. Also, the results have revealed a remarkable achievement in the security and stability of the protection scheme with an automatic resetting operation. Under the influence of the different internal and external faults, the evaluated percentages of the dependability (DR), security (SR), reliability (RR), and accuracy (AR) of the protection algorithm surpass 99.40%. Besides, it is a proper solution for avoiding a relay operation delay in the event of severe internal faults because of the harmonic content (resulting from the DC components or the CT saturation extent) inserted in the fault currents. Additionally, it can be used to protect different power system components with different voltage and power ratings in both conventional and smart grids. Also, the speed of the relay operation can be adjusted by altering the size of the data set, which can be selected within a single cycle. In the suggested approach method, the window length has been the primary determinant of the total post-fault data duration needed to detect a fault. The algorithm has required approximately 10 ms of the current signal following the fault inception time to make a reliable detection decision. Consequently, the proposed technique has a response time of approximately 10 ms in the case of internal faults. This time is suitable for avoiding the operation of the proposed scheme in the situation of non-fault or external fault disturbances. Therefore, it provides a quick response time to avoid the probability of CT saturation presence and its detrimental consequences.

## Electronic supplementary material

Below is the link to the electronic supplementary material.

Supplementary Material 1

## Data Availability

All data generated or analysed during this study are included in this published article [and its supplementary information files].

## References

[1] Jena, S. & Bhalja, B. R. Sampled value-based bus zone protection scheme with dq-components. *IET Gener. Transm. Distrib.* **14**(20), 4520–4528 (2020).

[2] Wu, H., Dong, X. & Wang, Q. New principle of busbar protection based on a fundamental frequency polarity comparison. *PLoS ONE* **14**(3), e0213308 (2019). 30897115 10.1371/journal.pone.0213308 PMC6428346

[3] Jena, S. & Bhalja, B. R. Initial travelling wavefront-based bus zone protection scheme. *IET Gener. Transm. Distrib.* **13**(15), 3216–3229 (2019).

[4] Hossain, M., Leevongwat, I. & Rastgoufard, P. Design and testing of a bus differential protection scheme using partial operating current (POC) algorithm. *Electr. Power Syst.* **157**, 29–38 (2018).

[5] Hossain, M., Leevongwat, I. & Rastgoufard, P. Partial operating current characteristics to discriminate internal and external faults of differential protection zone. *IET Gener. Transm.Distrib.* **12**(2), 379–387 (2018).

[6] Qais, M., Khaled, U. & Alghuwainem, S. Improved differential relay for busbar protection scheme with saturated current transformers based on second order harmonics. *J. King Saud Univ. Eng. Sci.* **30**(4), 320–329 (2018).

[7] Jena, S. & Bhalja, B. R. Numerical busbar differential protection using generalized alpha plane. *IET Gen. Trans Dis.* **12**, 227–234 (2018).

[8] Gil, M. & Abdoos, A. A. Intelligent busbar protection scheme based on combination of support vector machine and S-transform. *IET Gen. Trans. Dis.* **11**, 2056–2064 (2017).

[9] Ristanovic, D. et al. Bus differential protection in industrial systems with generators connected directly to the main distribution bus. *IEEE Trans. Ind. Appl.* **52**(4), 3574–3583 (2016).

[10] Chothani, N. G. & Bhalja, B. R. A new algorithm for busbar fault zone identification using relevance vector machine. *Elect. Power Compon. Syst.* **44**, 193–205 (2016).

[11] Song, S. & Zou, G. B. A novel busbar protection method based on polarity comparison of superimposed current. *IEEE Trans. Power Deliv.* **30**, 1914–1922 (2015).

[12] Allah, R. A. Adaptive busbar differential relaying scheme during saturation period of current transformers based on alienation concept. *IET Gener. Transm. Distrib.* **10**(15), 3803–3815 (2016).

[13] R. Abd Allah, 2016 “Adaptive relay scheme with dual protection techniques based on differential and alienation principles”, International Organization of Scientific Research-Journal of Electronics and Electrical Engineering (IOSR-JEEE), E-ISSN: 2278–1676, p-ISSN: 2320–3331 Vol. 11(1) IV 33–56.

[14] Abd Allah, R., Moussa, S., Shehab-Eldin, E. H. & Hamed, M. N. G. Adaptive busbar differential protection based on current transformer saturation degrees. *Eng. Res. J. (ERJ)* **33**(3), 227–232 (2010).

[15] Oliveira, M. O., Bretas, A. S. & Ferreira, G. D. Adaptive differential protection of three-phase power transformers based on transient signal analysis. *Int. J. Electr. Power Energy Syst.* **57**, 366–374 (2014).

[16] An, W. et al. An adaptive differential protection and fast auto-closing system for 10 kV distribution Networks Based on 4G LTE Wireless Communication. *Future Internet* **12**(1), 2 (2020).

[17] Sarangi, S. & Pradhan, A. K. Adaptive α-plane line differential protection. *Int. J. Inst Eng. Technol.* **11**(10), 2468–2477 (2017).

[18] Lin, H. et al. Adaptive protection combined with machine learning for microgrids. *IET Gener. Transm. Distrib.* **13**(6), 770–779 (2019).

[19] Muda, H. & Jena, P. Superimposed adaptive sequence current based microgrid protection: A new technique. *IEEE Trans. Power Deliv.* **32**(2), 757–767 (2017).

[20] Bukhari, S. et al. A protection scheme for microgrid with multiple distributed generations using super imposed reactive energy. *Int. J. Electr. Power Energy Syst.* **92**, 156–166 (2017).

[21] Shuanghui, Wu. An adaptive limited wide area differential protection for power grid with micro sources. *Prot. Control Mod. Power Syst.* **2**(21), 1–9 (2017).

[22] Ahmad, A., Othman, M. L., Zainab, K. K. & Hizam, H. Adaptive ANN based differential protective relay for reliable power transformer protection operation during energisation. *IAES Int. J. Artif. Intell.* **8**(4), 307–316 (2019).

[23] Dr. O. V. Ganana Swathika, S. Angalaeswari, V. Anantha Krishnan, Dr. K. Jamunaa , Dr. J.L. Febin Daya: “Fuzzy decision and graph algorithms aided adaptive protection of microgrid”, *1*^*st*^* International Conference on Power Engineering, Computing and Control, PECCON-2017, 2- 4 March 2017*, VIT University, Chennai Campus, Energy Procedia 117, 2017, pp. 1078–1084.

[24] Panahi, M. S. P., Azarakhsh, J. & Raisi, Z. A novel method in differential protection of power transformer using wavelet transform and correlation factor analysis. *Bull. Soc. Roy. Sci. Liège* **85**, 1119–1135 (2016).

[25] Seyedi, H. & Asghari Govar, S. Adaptive CWT-based transmission line differential protection scheme considering cross-country faults and CT saturation. *IET Gener. Transm. Distrib* **10**(9), 2035–2041 (2016).

[26] Elbana, M. S., Abbasy, N., Meghed, A. & Shaker, N. µPMU-based smart adaptive protection scheme for microgrids. *J. Mod. Power Syst. Clean Energy* **7**(4), 887–898 (2019).

[27] Shah, A. M. A new adaptive differential protection scheme for tap changing power transformer. *Int. J. Emerg. Electr. Power Syst.* **16**(4), 339–348 (2015).

[28] Manufacture’s Catalogue of the Siemens SIP: “Protection, control, measurement, and automation functions”, SIPROTEC 4 – Devices, Siemens SIP · Edition No. 8/ 8.1, Chapter 9: Busbar Differential Protection/7SS52, Sep 4, 2020.

[29] Mahmoud, R. A., Ghaly, K. G. & Hasan, S. A. Adaptive overcurrent protection considering fault current limiters effect. *Int. J. Sci. Rep.* **15**, 20146. 10.1038/s41598-025-05135-5 (2025). 10.1038/s41598-025-05135-5 PMC12181245 40542036

[30] Mahmoud, R. A. & Malik, O. P. Adaptive overcurrent relay (AOCR) based on Fano Factors of current signals. *IET J. Eng.* **2023**(4), e12267. 10.1049/tje2.12267 (2023).

[31] Sadeghi, S., Naghshbandy, A. H., Moradi, P. & Bagheri, A. Optimal adaptive coordination of overcurrent relays in power systems protection using a new hybrid metaheuristic algorithm. *IET Renew. Power Gener.* **18**, 1948–1971. 10.1049/rpg2.13041 (2024).

[32] Koloushani, S. M. & Taher, S. A. A new virtual consensus-based wide area differential protection. *IET Gener. Transm. Distrib.* **18**, 1906–1918. 10.1049/gtd2.13168 (2024).

[33] Koloushani, S. M. & Taher, S. A. Dynamic wide-area cooperative protection: A new approach. *IET Gener. Transm. Distrib.* **17**, 5198–5211. 10.1049/gtd2.13030 (2023).

[34] Joband, M., Homayounifar, S. & Sarlak, M. A superimposed current based busbar protection scheme using slope degree of grey incidence analysis model. *IET Gener. Transm. Distrib.* **17**, 161–180. 10.1049/gtd2.12671 (2023).

[35] Mahmoud, R. A. Integrated busbar protection scheme utilizing a numerical technique based on coherence method. *IET J. Eng.* **2022**(1), 94–119. 10.1049/tje2.12100 (2022).

[36] Kai, Li., Mengshu, Li. & Zhengfeng, L. Analytical closed-form expressions of DC current ripple for three-level neutral point clamped inverters with space-vector pulse-width modulation. *IET Power Electron.* **9**(5), 930–937 (2016).

[37] Dayal, R., Dwari, S. & Parsa, L. Design and implementation of a direct AC-DC boost converter for low voltage energy harvesting. *IEEE Trans. Ind. Electron.* **58**(6), 2387–2396 (2011).

[38] Mahmoud, R. A. Detection and assessment scheme of voltage and current unbalance for three phase synchronous generators using dual numerical techniques based on correlation and deviation percentage concepts. *IET J. Eng.*10.1049/tje2.12051 (2021).

[39] Mahmoud, R. A. & Elwakil, E. S. Experimental investigations using quadratic-tripping characteristics based on alienation/coherence coefficients of voltage and current signals for synchronous generators protection. *IET Gener. Transm. Distrib.* **15**(21), 2978–3000 (2021).

[40] Mahmoud, R. A. Experimental performance verification of an intelligent detection and assessment scheme for disturbances and imbalances of three-phase synchronous machine output using coherence estimators. *Int. J. Sci. Rep.* **14**, 26278 (2024). 10.1038/s41598-024-76343-8 PMC11530546 39487212

[41] Adly, A. R. et al. Directional protection scheme using impedance approach for transmission lines. *Int. J. Sci. Rep.* **15**, 20217 (2025). 10.1038/s41598-025-06609-2 PMC12181255 40542069

[42] Mahmoud, R. A. Experimental evaluation of differential voltage protection scheme based on a coherence function applied to AC machine stator windings. *Int. J. Sci. Rep.* **15**, 12399 (2025). 10.1038/s41598-025-93210-2 PMC11992023 40216807

[43] Mahmoud, R. A. Experimental performance examination of a coherence technique-based numerical differential current relay for AC machine stator windings protection. *Int. J. Sci. Rep.* **15**, 7751 (2025). 10.1038/s41598-025-89092-z PMC11882915 40044710

[44] Mahmoud, R. A. Digital protection scheme based on Durbin Watson and Pearson similarity indices for current signals practically applied to power transformers. *Int. J. Sci. Rep.* **15**, 12214 (2025). 10.1038/s41598-025-91491-1 PMC11985498 40210763

[45] Elsadd, M. A., Yousef, W. & Abdelaziz, A. Y. New adaptive coordination approach between generator-transformer unit overall differential protection and generator capability curves. *Electr. Power Energy Syst.* **118**, 1–15 (2020).

