# Supplementary material for: Adaptive differential current relay based on form/ripple factors for busbar current signals
Source: Sci Rep. 2025 Aug 12;15:29552. doi: 10.1038/s41598-025-12832-8 (PMC12344150; doi:10.1038/s41598-025-12832-8)
Supplement: Supplementary file 1 — Supplementary Material 1 [file 41598_2025_12832_MOESM1_ESM.pdf]

Nomenclatures:

Symbols	Abbreviations	Symbols	Abbreviations
N_c	The number of circuits connected to the protected element, (the protected busbar is connected to $N_c = 4$ feeders),	K_{s3}	The tripping characteristics slope based on the form factor FF_i,
$i_{ds}(k)$	The estimated differential current at '*k*' sample index for '*S*' phase of the protected element,	K_{s4}	The tripping characteristics slope based on the ripple factor RF_i,
$i_{rs}(k)$,	The estimated restraining current at '*k*' sample index for '*S*' phase of the protected element,	c_1, c_2, c_3 and c_4	Constants,
$i_{sm}(k)$	The measured secondary current at '*k*' sample index for '*S*' phase obtained from the installed CT of a circuit '*m*'.	c_5, c_6, c_7 and c_8	Constants,
m	The index of the circuit order (m = 1 to N_c),	K_{sMIN} and K_{sMAX}	The minimum and maximum slopes of the protective relay characteristics,
I_{ds}	The amplitudes of the fundamental components of $i_{ds}(k)$,	F_1	The location of external fault (close to busbar BB_1),
I_{rs}	The amplitudes of the fundamental components of $i_{rs}(k)$,	F_2	The location of internal fault on the busbar BB_1,
I_{dthr}	The sensitive differential current for monitoring CTs faults,	Θ	The fault inception angle,
I_{do}	The lower threshold (pickup) differential current of the protected element,	α	The power system angle,
k	The index of the sample order,	ω	The angular velocity of the power system ($\omega = 2 \pi f$),
K_s	The restraint factor (slope) of each differential relay characteristic,	R_f	The fault resistance imposed from the faulted point to the ground point in case of ground fault, or the fault resistance inserted between the two faulted phases in case of phase fault,
T_d	The time-to-saturation, it is the delay time from the fault time inception to CT saturation starting,	*ATP*	Alternative Transient Program,
$i_s(k)$	The instantaneous values of the measured current signal at sample '*k*' for '*S*' phase,	t_f	The fault inception time,
I_{sRMS}	The RMS value of the measured current signal ($i_s(k)$) for '*S*' phase,	k_f	The sample index at which the fault occurs,
I_{sDC}	The average value (i.e. DC component) of the measured current signal ($i_s(k)$) for '*S*' phase,	N_{sim}	The total number of samples per the simulation time,
I_{sacRMS}	The effective RMS value of AC component in	*SLG*	Single Line-to-Ground,

	the measured current signal for '*S*' phase,		
FF_i	The form factor estimated for current signal, it is the ratio of RMS value to the DC (average) value of the measured current,	*A-G*	The fault located between the '*A'* phase and the ground point,
RF_i	The ripple factor estimated for current signal, it is the ratio of the effective RMS value of the AC component (ripple component) to the DC value of the measured current,	*DLG*	Double Line-to-Ground,
Δ	The deviation of threshold value for the ripple factor RF_i, (Δ= 0.1 used in the proposed algorithm),	*DL*	Double Line,
RF_x	The setting value of the ripple factor RF_i, (RF_x= 1.21+ Δ=1.31 used in the proposed algorithm),	*3LG*	Three Line-to-Ground,
FF_{ix}	It is the ratio of RMS value to the RMS value of the AC component of the measured current,	*AC component*	Alternating current component,
RF_{ix}	It is the ratio of the effective value of DC component (ripple component) to the RMS value of the AC component of the measured current,	*DC component*	Direct current component,
F_m	The maximum first derivative obtained from the stored pre-fault current,	V_{1Max}	The peak phase voltage of synchronous generator,
Fs	The setting value of first derivative for the secondary current ($i_{sm}(k)$),	V_{2Max}	The peak phase voltage of the electrical network,
$F_{ism}(k)$	The first derivative for the secondary current ($i_{sm}(k)$) of each CT in the circuit '*m'* for '*S*' phase,	F_{1op}	The operating frequency of synchronous generator,
$i_s(k)$	The measured secondary current signal (i_s), at sample index *k*, for phase '*S*',	F_{2op}	The operating frequency of the electrical network,
$i_s(k-1)$	The measured secondary current signal (i_s), at sample index (*k-1*), for phase '*S*',	δ_1	The operating power angle of synchronous generator,
h	The sampling time interval, ($h = 1/ f_s$),	δ_2	The operating power angle of the electrical network,
f_s	The sampling frequency of the system (f_s= 5 kHz used in the proposed algorithm),	R_n	The generator grounding impedance through the neutral point,
S	The phase designation *A*, *B*, or *C*,	V_n	The nominal voltage of the power system,
$DF_s(k)$	The instantaneous value of directionality factor calculated using the input and output currents of each *'S'* phase of the protected equipment,	I_n	The nominal current of the power system,
DF_a, DF_b and	The instantaneous value of directionality factors for the three phases *A*, *B*, and *C*, *respectively*,	R_b	The current transformer burden,

DF_c			
DF_{sv}	The average value of directionality factor calculated using the input and output currents for each data window of each *'S'* phase of the protected equipment,	R_{CT}	The resistance of current transformer secondary winding,
DF_{av}, DF_{bv} and DF_{cv}	The average value of directionality factors for the three phases *A*, *B*, and *C*, *respectively*,	R_{lead}	The lead resistance connected between the current transformer terminals and the relay,
N_s	The number of samples per cycle used in the simulation (N_s = 100 Samples/Cycle),	*SLD*	Single Line Diagram,
N_w	The number of samples per the selected data window size (N_w = 50 Samples/Cycle),	*TL*	Transmission Line,
i_{sin}	The summation of the input currents for the protected equipment,	*CT*	Current Transformer,
i_{sout}	The summation of the output currents for the protected equipment,	*CTR*	Current Transformer Ratio,
K_{s1}	The tripping characteristics slope based on the form factor FF_{ix},	*BB*	Busbar,
K_{s2}	The tripping characteristics slope based on the ripple factor RF_{ix},	*CB*	Circuit Breaker,

Appendix 1: The parameter's data of the simulated power system components

The parameter of the power system component	Data
**<u>Synchronous generator (Sending source):</u>**	
Rated Volt-ampere	*320 MVA*
Rated line voltage	*19.57 kV*
voltage phasor angle	30^0
Rated frequency	*50 Hz*
Number of poles	*2*
Neutral grounding impedance (R_n)	*0.77 Ω*
**<u>Power Network (Receiving source):</u>**	
Nominal line voltage	*500kV*
Voltage phasor angle	0^0
Nominal frequency	*50 Hz*
Volt-ampere short circuit	*25 GVA* ($i_{s.c}$ = 10 kA)
**<u>Transmission Lines (1&2):</u>**	
Positive sequence R	*0.0217 Ω /km*
Zero sequence R	*0.247 Ω /km*
Positive sequence XL	*0.302 Ω /km*
Zero sequence XL	*0.91 Ω /km*
Positive sequence 1/Xc	*3.96 μ℧ /km*
Zero sequence 1/Xc	*2.94 μ℧ /km*
Transmission line long (Km)	*100 Km*
**<u>Aux. Load (load 1):</u>**	*10.85 + j6.72 at PF = 0.85 lag*
**<u>Main Load (load 2):</u>**	*1.25 + j0.75 at PF = 0.85 lag*
**<u>Main Load (load 3):</u>**	*1.25 + j0.75 at PF = 0.85 lag*

**Current Transformer (CT):**	
CTR	*12000/1*
Rated burden	*30 VA*
Class	*5p20*

Appendix 2: The input data of the proposed protection algorithm

Quantity designation	Quantity description	Input data
$i_{a1m}(k)$, $i_{b1m}(k)$ and $i_{c1m}(k)$	The current measurements of *a*, *b* and *c* phases, respectively, at the instant '*k'* taken for the incoming feeder '*m*' at the input terminal of the protected busbar,	They are measured using the ATP software
$i_{a2m}(k)$, $i_{b2m}(k)$ and $i_{c2m}(k)$	The current measurements of *a*, *b* and *c* phases, respectively, at the instant '*k'* taken for the outgoing feeder '*m*' at the output terminal of the protected busbar,	
F_c	The fundamental frequency of the current signals	50 *Hz*
T_c	The cycle time interval	20 milliseconds
F_{sp}	The frequency rate of the digital system for the current signals	5.0 *kHz*
T_{sp}	The sampling time	0.2 milliseconds
N_s	The sample size per single cycle, $N_s = T_c / T_{sp}$ or $N_s = F_{sp} / F_c$	100 samples/cycle
N_w	The sample size per data window, $N_w = N_s/2$	50 samples/data window
T_{ds}	The full simulation time	0.3 seconds = 15 cycles
N_{sim}	The total number of samples per the full simulation time	1500 samples
F_s	The setting value of the first derivative, where, F_m is the maximum first derivative obtained from the stored pre-fault current.	200% F_m
$DF_{setting}$	The setting value of the directionality factor	1.0
RF_x	The ripple factor limit	It is based on the selected data window size (as given in Table 1)
FF_x	The form factor limit	
I_n	The busbar nominal current	1.0 per unit
I_{do}	The pickup differential current	0.2
K_{sMIN}	The minimum slope of the characteristic curve	0.1
K_{sMAX}	The maximum slope of the characteristic curve	1.0
